# Age-Specific ADHD and Internalizing/Externalizing Comorbidity in Children with Neurofibromatosis Type 1: A Multi-Site Study

**DOI:** 10.3390/cancers18030529

**Published:** 2026-02-06

**Authors:** Dan Liu, Pamela L. Wolters, Bonita P. Klein-Tasman, Karin S. Walsh, Jonathan M. Payne, Natalie Pride, Stephanie M. Morris, Yang Hou

**Affiliations:** 1 Department of Behavioral Sciences and Social Medicine, College of Medicine, Florida State University, Tallahassee, FL 32306, USA; dan.liu@med.fsu.edu; 2 Pediatric Oncology Branch, Center for Cancer Research, National Cancer Institute, National Institutes of Health, Bethesda, MD 20892, USA; woltersp@mail.nih.gov; 3 Department of Psychology, University of Wisconsin–Milwaukee, Milwaukee, WI 53211, USA; bklein@uwm.edu; 4 Children’s National Hospital, Washington, DC 20010, USA; kwalsh@childrensnational.org; 5 Department of Psychiatry & Behavioral Health, George Washington University School of Medicine, Washington, DC 20037, USA; 6 Murdoch Children’s Research Institute, Melbourne 3052, Australia; jonathan.payne@mcri.edu.au; 7 Department of Paediatrics, University of Melbourne, Melbourne 3010, Australia; 8 Children’s Hospital at Westmead, Sydney 2145, Australia; natalie.pride@health.nsw.gov.au; 9 Kennedy Krieger Institute, Baltimore, MD 21211, USA; morrisst@kennedykrieger.org

**Keywords:** neurofibromatosis type 1, age-varying associations, ADHD, mental health, familial versus sporadic NF1

## Abstract

**Objective**: The current study tested (1) how ADHD symptoms and internalizing or externalizing problems covaried across ages 3–18 in children with neurofibromatosis type 1 (NF1), and (2) whether demographic and NF1-specific factors moderated the associations. **Method**: We analyzed integrated cross-sectional data of 685 observations from 455 children and adolescents with NF1 (*M*_age_ = 9.79 years, *SD* = 3.88; 43% female) across six institutions in the United States and Australia. ADHD symptoms (inattention and hyperactivity/impulsivity) and internalizing/externalizing problems were assessed via parent-report measures. Time-varying effect modeling was employed to examine the age-specific associations between ADHD symptoms and internalizing/externalizing problems. Moderation analyses tested effects of sex, parental education, and NF1 inheritance mode (familial vs. sporadic). **Results**: Inattention and hyperactivity/impulsivity symptoms were associated with greater internalizing and externalizing problems across ages 3–17. Inattention links were similar across ages, while the hyperactivity/impulsivity-externalizing link was stronger in early childhood than during adolescence. NF1 inheritance mode significantly moderated the inattention-externalizing link, with stronger associations observed among children with familial NF1. Other moderators were nonsignificant. **Conclusions**: ADHD symptoms are robustly linked to internalizing and externalizing problems from childhood to middle adolescence in children with NF1, with familial NF1 emerging as a potentially elevated risk factor. Future longitudinal and experimental research is needed to inform integrated intervention approaches, especially for those with familial NF1.

## Simple Summary

Children with neurofibromatosis type 1 (NF1) often experience attention problems and difficulties with emotions and behavior, but little is known about how these challenges covary with age. In this study, we analyzed data of 685 observations from 455 children with NF1, ages 3 to 18, to examine how attention-deficit/hyperactivity symptoms relate to emotional and behavioral problems across childhood and adolescence. We found that inattention and hyperactivity/impulsivity symptoms were consistently linked to higher levels of internalizing problems (like anxiety and depression) and externalizing problems (like aggression). The connection between hyperactivity/impulsivity and externalizing problems was strongest in younger children. Children with a family history of NF1 showed a stronger link between inattention and externalizing problems. These findings highlight the importance of monitoring attention and behavioral difficulties in children with NF1 and suggest that children with familial NF1 may benefit from early, targeted support.

## 1. Introduction

Neurofibromatosis type 1 (NF1) is a common autosomal dominant neurogenetic disorder affecting approximately 1 in 3000 individuals [1]. Although typically defined by dermatological and neurological features (e.g., café-au-lait macules, Lisch nodules, optic pathway gliomas, and neurofibromas), NF1 is also related to a broad range of neurodevelopmental and psychiatric difficulties [2,3]. Impairments in executive function, including attentional control, working memory, and inhibitory control, are frequently observed in children with NF1 [4] and may contribute to elevated risks for multiple emotional and behavioral problems. Among these, attention-deficit/hyperactivity disorder (ADHD) is one of the most commonly diagnosed conditions in affected children [5,6]. Internalizing problems (e.g., anxiety, depression) and externalizing problems (e.g., aggression, rule-breaking) are also more pronounced in NF1, compared with typically developing peers [7]. These difficulties often co-occur and compound functional impairments across academic, social, and family contexts. Despite growing awareness of these challenges, the developmental course of and interrelations among ADHD symptoms and internalizing or externalizing problems in NF1 remain poorly understood, limiting current treatment approaches.

ADHD is a neurodevelopmental disorder characterized by developmentally inappropriate levels of inattention and/or hyperactivity-impulsivity symptoms that interfere with daily functioning [8]. The ADHD symptom domains are clinically and functionally distinct and often exhibit different developmental trajectories, with substantial interindividual variability [9,10,11]. For example, a population-based, longitudinal twin study found that inattention symptoms generally increased while hyperactivity/impulsivity symptoms generally decreased from childhood to middle adolescence [11]. They also found that individuals shifted in ADHD subtypes over time, with some shifting from a combined type (elevated levels of both inattention and hyperactivity/impulsivity) to an inattentive type while some others shifting from a hyperactive/impulsive type to a combined type. These developmental changes may reflect underlying shifts in brain maturation, particularly in the prefrontal cortex and associated executive function networks [12,13].

Similar developmental patterns have been observed for internalizing and externalizing problems. Externalizing behaviors tend to become more prominent from childhood to middle adolescence, often declining during late adolescence as behavioral regulation improves [14,15]. Internalizing symptoms typically follow the opposite trajectory: They are often less visible in early childhood but increase during adolescence, particularly among females [16]. These shifts may reflect both neurobiological maturation and cognitive vulnerability towards increased exposure to emotional and social stressors [17,18].

ADHD symptoms rarely occur in isolation. Comorbidity with internalizing and externalizing problems is well documented and highly prevalent in both clinical [19] and non-clinical populations [20,21,22]. Inattention has been more closely linked to internalizing problems [23], possibly due to its associations with poorer adjustment in other domains that are commonly associated with internalizing problems, such as academic underachievement [24]. Conversely, hyperactivity and impulsivity are more strongly associated with externalizing behaviors such as conduct problems and oppositional defiant disorder [23], which may arise from poor inhibitory control and increased emotional reactivity [25]. These associations are dynamic and may vary across developmental stages, although current research on the age-varying associations is scarce. Preliminary evidence suggests that the correlation between ADHD symptoms and externalizing problems increase over time from ages 8–9 to ages 19–20 [26], which also suggests a more salient association transitioning from mid adolescence to adulthood than from childhood to early adolescence. Another study found that individuals with elevated ADHD symptoms exhibited different trajectories of anxiety and depression, with some showing increasing risks over time [27]. The co-occurrence of these difficulties further exacerbates functional impairments across academic, social, and other developmental domains [28] and is, therefore, particularly harmful for affected individuals.

In the context of NF1, these developmental and comorbidity patterns may be amplified or qualitatively altered by disease-specific factors. Neurobiological mechanisms related to NF1 variants, including disruptions in neurofibromin and Ras/MAPK signaling important for neural development and executive functioning, may increase vulnerability to attentional and behavioral dysregulation across development [1,2]. Concurrently, psychosocial stressors associated with managing a chronic, rare condition may further shape the emergence and co-occurrence of internalizing and externalizing symptoms over time [29]. Although elevated levels of ADHD symptoms [6] and internalizing/externalizing problems [7] in NF1 have been consistently reported, important gaps remain in our understanding of how these symptoms co-occur across development. Existing studies have often been constrained by relatively small sample sizes, single-site designs, and cross-sectional or age-aggregated analytic approaches [30], limiting the ability to examine whether associations between ADHD symptom dimensions and internalizing/externalizing problems vary across childhood and adolescence. Addressing these limitations requires larger multisite samples and analytic methods capable of characterizing developmental heterogeneity in symptom co-occurrence.

Furthermore, it remains unclear whether these associations are moderated by demographic or clinical variables such as sex, parental education, or mode of NF1 inheritance, which are all factors that may signal variation in risk. For example, evidence from a national U.S. sample of 9–10-year olds suggests that the links between a history of ADHD diagnosis and internalizing or externalizing problems were stronger among males than females [31]. Despite a lack of research on the moderating role of parental education, such an effect is plausible given consistent findings showing that children from more highly educated households tend to exhibit lower levels of ADHD symptoms [32] and fewer internalizing or externalizing problems [33]. Similarly, the mode of NF1 inheritance may moderate these associations, as familial NF1 has been linked to more severe neurocognitive profiles [34]. However, research has rarely examined how these variables influence the age-related patterns of ADHD comorbidity in NF1.

To address these gaps, the present study employed integrative data analysis (IDA) to pool data of 685 observations from 455 children and adolescents with NF1 across six institutions in the United States and Australia. Using time-varying effect modeling (TVEM), an innovative statistical technique for capturing potential non-linear age patterns using cross-sectional data [35], we examined how ADHD symptoms of inattention and hyperactivity/impulsivity were associated with internalizing and externalizing problems across ages 3 to 18 (Aim 1). In addition, we tested whether these associations varied by sex, parental education, and mode of NF1 inheritance (Aim 2). By modeling these associations across ages, this study aims to clarify the age-related patterns of ADHD-internalizing/externalizing comorbidity in NF1 and identify potential windows and strategies for early identification, prevention, and intervention.

## 2. Materials & Method

### 2.1. Participants and Procedure

The current study utilized cross-sectional data of 685 observations from 455 children and adolescents with NF1 collected across six independent institutions in the United States and Australia (Table S1). The anonymized dataset was combined through integrative data analysis (IDA) [36], a methodological framework that aggregates datasets from multiple sources to enhance statistical power and improve diversity across demographic variables [37]. All analyses were performed at the observation level; accordingly, the reported sample sizes (*n*) reflect the number of observations unless otherwise specified. Institutional Review Board (IRB) approval was obtained at all participating sites. Approximately half of the institutions collected written informed consent from participants or guardians, while others contributed retrospective clinical records. The overarching IDA protocol received ethical approval at the University of Kentucky (ID 67554) and Florida State University (ID STUDY00003513).

### 2.2. Measures

**Outcomes**. Internalizing and externalizing problems were assessed using parent-rated versions of either the Child Behavior Checklist (CBCL; *n* = 209, 31%) [38] or the Behavior Assessment System for Children (BASC; *n* = 476, 69%) [39,40]. The internalizing composite included anxious/depressed, withdrawn/depressed, and somatic complaints in CBCL, and included anxiety, depression, and somatization in BASC. The externalizing composite included aggressive and rule-breaking behaviors in CBCL, and hyperactivity, aggression, and conduct problems in BASC. The CBCL and the BASC are two of the most widely used standardized measures of emotional and behavioral problems in the NF1 population [7]. Both measures have demonstrated robust psychometric properties, including strong validity and consistency across emotional and behavioral domains [41]. The internalizing and externalizing measures used at each site are presented in Table S1. Analyses were conducted using standardized T scores (*M*_norm_ = 50, *SD_norm_* = 10), with higher values indicating greater symptom severity.

**Focal covariates**. ADHD symptoms, including inattention and hyperactivity/impulsivity were assessed using parent-rated Conners Rating Scales (CRS) and the ADHD Rating Scales (See descriptive information of each site in Table S1). Five different versions of Conners Rating Scale were used: Conners-3 Parent Report (Conners 3-P) [42], Conners-3 Parent Report Short Form [42], Conners’ Parent Rating Scales-Revised (CPRS-R) [43], Conners’ Parent Rating Scale-Revised Short Form [44], Conners ADHD/DSM-IV Scales [44]. ADHD Rating Scale-IV: Home Version (ADHD RS-IV) [45] was used at one site. Convergent validity has been demonstrated for the various versions of the CRS with strong correlations between matching subscales (*r* = 0.66–0.74) [42]. Additionally, moderate to strong correlations have been found between the CRS and the ADHD Rating Scale-IV (*r* = 0.53–0.73) [46], supporting their alignment in measuring ADHD-related symptoms.

**Background information**. Demographic and NF1-specific factors were reported by parents or primary caregivers or retrieved from medical records (Table S2). Variables included in analyses were participants’ age (in years); biological sex (categorized as *male* and *female*); parental education, which was initially coded into five categories based on information provided by each site: (1) less than high school, (2) high school or partial high school, (3) some college, community college, or associate degree, (4) college or university degree, and (5) graduate or professional training, and then further categorized as *low* [i.e., lower than college] and *high* [i.e., some college or above] for visualization in subgroup analysis); mode of NF1 inheritance (whether NF1 was inherited or not: *familial NF1* vs. *sporadic NF1*); and whether plexiform neurofibromas were present (*yes* or *no*).

### 2.3. Statistical Analysis

TVEM with the P-spline approach was used to delineate the associations between ADHD symptoms (i.e., inattention and hyperactivity/impulsivity) and internalizing or externalizing problems across ages, following guidelines outlined in Tan et al. (2012) [35]. This technique, known for its flexibility in modeling developmental trends without imposing parametric constraints [35], is well-suited to capturing dynamic developmental processes across time or ages. Although originally designed for longitudinal data analysis [47], this technique has been adapted for cross-sectional data to explore age-related trends [48]. A participant-level random intercept was included to account for within-subject correlation arising from repeated observations for some participants.

For Aim 1, we used TVEM to test the associations between inattention or hyperactivity/impulsivity and internalizing or externalizing problems across ages 3 to 18. For Aim 2, we examined the moderating effects of sex, parental education, and mode of NF1 inheritance using a two-step approach: First, the interaction term between each ADHD variable and each moderator was included within the TVEM models; second, when a statistically significant interaction term emerged at any age point, we conducted stratified analyses for subgroups of each moderator, to aid the interpretation of moderation patterns [48,49]. The three moderators were examined in separate models, rather than included simultaneously, to preserve model interpretability and stability. Estimates were visualized as smooth functions of age with 95% confidence intervals. Associations were considered statistically significant when confidence intervals did not include zero. Non-overlapping 95% CIs between specific age points were considered as indicators of significant age differences. Analyses were conducted using a SAS macro (Version 3.1.1) [50], with missing cases excluded listwise from specific models.

### 2.4. Transparency and Openness

We report how we determined our sample size, all data exclusions, and all measures in the study, and we follow Journal Article Reporting Standards [51]. All data, analysis code, and research materials for this study are available on request from the corresponding author. This study’s design and its analysis were not pre-registered.

## 3. Results

### 3.1. Sample Characteristics

The combined sample included 685 observations from 455 children and adolescents with NF1, with a mean age of 9.79 years (*SD* = 3.88) ranging from 3 to18 years. The age distributions for each association in the total sample are presented in Table 1 and Table 2. Although sample sizes at individual ages varied (range = 22–69, except for age 18 with 0–6 participants), prior simulation work suggests that TVEM produces minimally biased estimates even with small numbers at some time points, as long as sufficient coverage exists across the age range [52,53]. The sample size for each association in main analyses (Aim 1) was larger than 15 for all age groups from 3 to 17, which were sufficient to yield reliable TVEM estimates and thus interpreted. The sample size for Step 1 of the moderation analyses (Aim 2) exceeded 15 across ages 3 to 17, supporting reliable detection of moderation effects at these ages. However, sample sizes for Step 2 analyses within each subgroup were small for certain age extremes. Accordingly, these subgroup results were intended to ease interpretation of overall patterns rather than to support formal statistical tests of group differences.

Among the participants who had background information available, 43.0% (*n* = 293/681) were females, 67.8% ( *n* = 375/553) had parents with some college or above education, 38.1% (*n* = 215/565) had familial NF1, and 69.2% (*n* = 225/325) had PNs. Sample characteristics by site and for the combined sample are presented in Table S1, with demographic and clinical details summarized in Table S2. Age-specific sample size distributions for the CBCL and BASC are shown in Table S3. Additional descriptive statistics and correlations of study variables are presented in Table S4.

### 3.2. Age-Varying Associations Between ADHD Symptoms and Internalizing/Externalizing Problems

**Figure 1:**
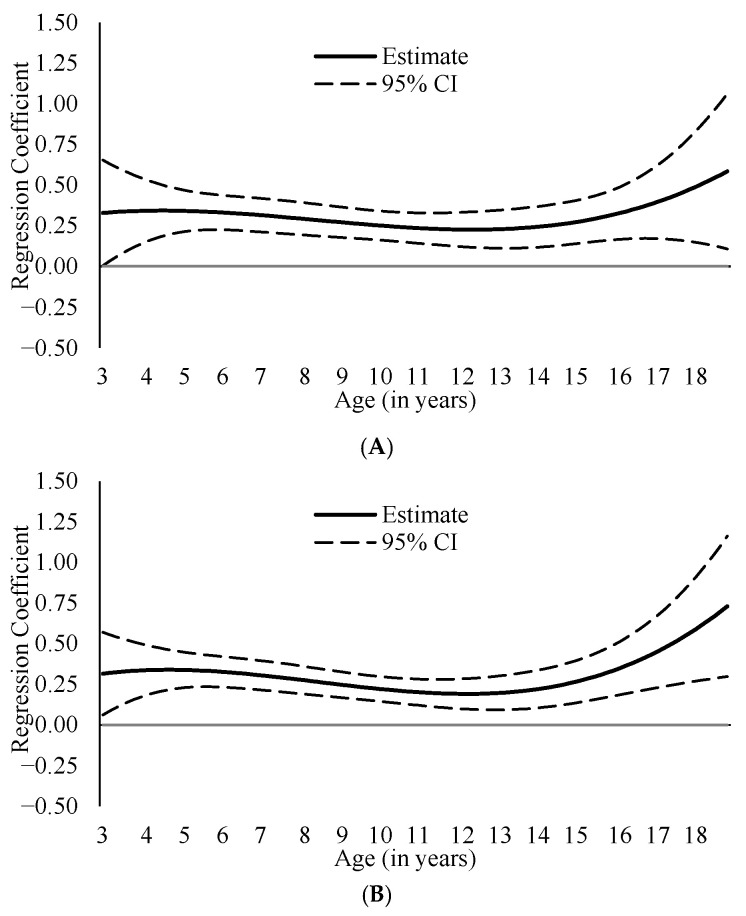
Age-Varying Associations between Attention Deficit/Hyperactivity Disorder Symptoms and Internalizing Problems. *Note*. These figures present age-specific associations between the following variables: (**A**). inattention and internalizing; (**B**). hyperactivity/impulsivity and internalizing. The *y*-axis represents regression coefficient estimates derived from T-score–scaled (*M* = 50, *SD* = 10) predictor and outcome. Statistically significant associations between variables are indicated by 95% confidence intervals (CIs) that do not include zero. Significant age differences are indicated by non-overlapping 95% CIs between specific age points.

**Figure 2:**
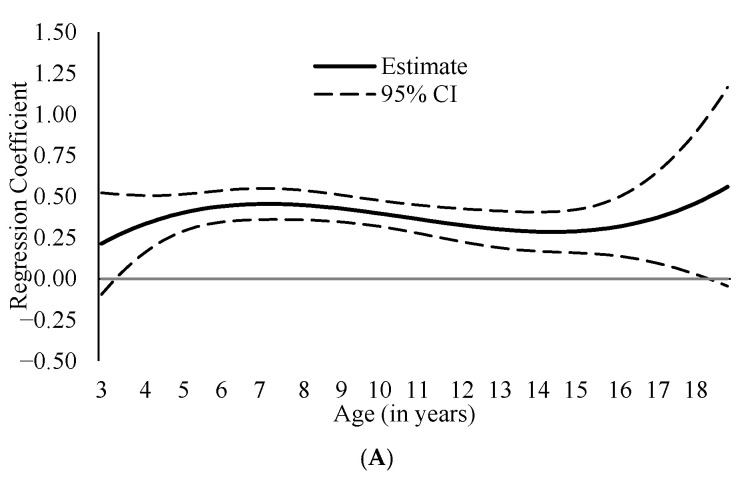

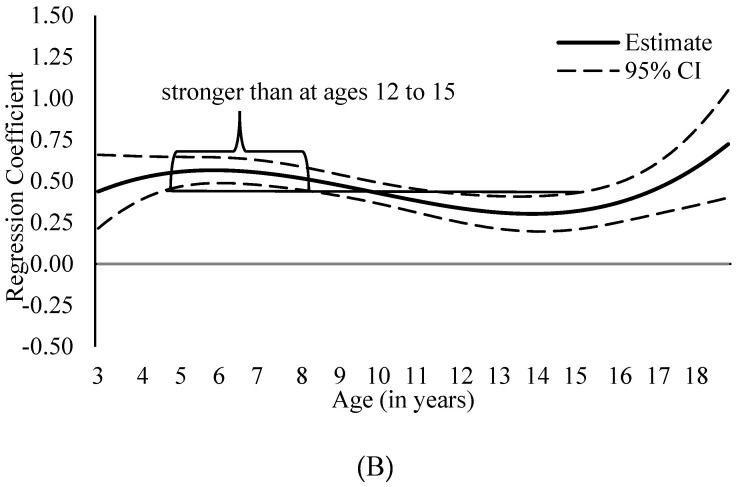
Age-Varying Associations between Attention Deficit/Hyperactivity Disorder Symptoms and Externalizing Problems. *Note.* These figures present age-specific associations between the following variables: (**A**). inattention and externalizing; (**B**). hyperactivity/impulsivity and externalizing. The *y*-axis represents regression coefficient estimates derived from T-score–scaled (*M* = 50, *SD* = 10) predictor and outcome. Significant associations between variables are indicated by 95% confidence intervals (CIs) that do not include zero. Significant age differences are indicated by non-overlapping 95% CIs between specific age points.

Both ADHD symptom dimensions—inattention and hyperactivity/impulsivity—were positively associated with internalizing (Figure 1; coefficient estimates presented in Table S5) and externalizing (Figure 2; coefficient estimates presented in Table S6) problems across ages 3–17, suggesting robust association between ADHD symptoms and internalizing/externalizing problems from early childhood to late adolescence. Because both predictors and outcomes were expressed as standardized T scores (*M* = 50, *SD* = 10), regression coefficients can be interpreted as standardized effect sizes, reflecting the expected standard deviation change in outcomes associated with a 1-SD increase in ADHD symptoms.

Associations between ADHD symptoms and internalizing problems were generally similar across ages, with inattention–internalizing coefficients ranging from β = 0.23 (95% CI [0.13, 0.33]) to β = 0.47 (95% CI [0.15, 0.79]) and hyperactivity/impulsivity–internalizing coefficients ranging from β = 0.19 (95% CI [0.10, 0.28]) to β = 0.56 (95% CI [0.26, 0.96]). Similarly, the association between inattention and externalizing problems showed limited age-related variation (βs = 0.21, 95% CI [−0.10, 0.52] to 0.44, 95% CI [0.04, 0.85]). In contrast, the association between hyperactivity/impulsivity and externalizing problems exhibited developmental variation, with stronger associations observed in younger children than in adolescents, ranging from β = 0.30 (95% CI [0.20, 0.41]) in early childhood (ages 5–8) to β = 0.57 (95% CI [0.49, 0.64]) in later childhood/adolescence (ages 12–15; Figure 2B).

### 3.3. Moderation Effects by Sex, Parental Education, and Mode of NF1 Inheritance

**Figure 3:**
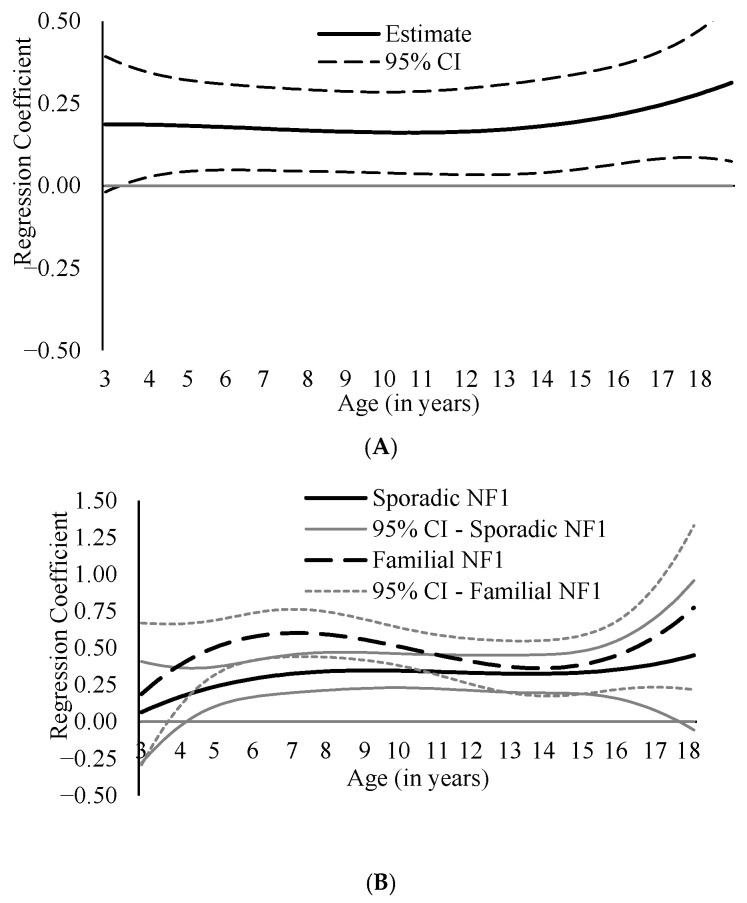
Inattention–Externalizing Link Moderated by the Mode of Neurofibromatosis Type 1 (NF1) Inheritance. *Note.* The following age-specific associations are presented: (**A**). interaction effect of inattention and NF1 inheritance mode on externalizing; (**B**). inattention and externalizing association across NF1 inheritance subgroups (solid lines for sporadic NF1 group, dashed lines for familial NF1 group). Significant associations between variables are indicated by 95% confidence intervals (CIs) that do not include zero.

Moderation analyses showed that the association between inattention and externalizing problems was significantly moderated by NF1 inheritance mode between ages 4 and 17, as the 95% confidence intervals for the interaction term were consistently above zero during this age range (Figure 3A). The follow-up subgroup analysis further revealed a larger coefficient estimate among children with familial NF1 at these ages (Figure 3B), compared with children with sporadic NF1. For example, at age 7, the association was stronger among children with familial NF1 (β = 0.60) than those with sporadic NF1 (β = 0.33), and at age 14, the association was weaker in both groups but remained larger in the familial group (familial: β = 0.36; sporadic: β = 0.33). None of the other tested moderation effects by sex, parental education, and mode of NF1 inheritance were statistically significant (Figures S1–S6).

### 3.4. Sensitivity Analyses

The analytic dataset consisted of repeated observations clustered within individuals, with individuals further grouped by study site. Intraclass correlation analyses revealed pronounced dependence at the participant level (ICC = 0.57–0.68 across models) and negligible clustering attributable to site (ICC = 0.0002–0.03). To assess whether accounting for site-level dependence affected results, we evaluated two TVEM configurations: a primary specification including random intercepts for participants only, and a secondary specification incorporating random intercepts for both participants and sites. Model comparisons were conducted using likelihood ratio tests with maximum likelihood estimation. The inclusion of site-level random effects did not lead to improved model fit (Table S7). Furthermore, prior simulation research indicates that when the number of clusters is small and cluster sizes are highly unequal—conditions present in the current study—adding site-level random effects may inflate standard errors and compromise the precision of variance component estimates [54]. Based on these considerations, the main results reported above were based on the participant-level random-intercept TVEM.

Additional sensitivity analyses were conducted to assess potential effects of measurement instrument differences (CBCL vs. BASC). The inclusion of instrument type as a covariate did not meaningfully alter the estimated age-varying associations (Figures S7 and S8). This suggests that the primary findings were robust to measurement differences, although residual instrument-related variability cannot be fully ruled out.

## 4. Discussion

This study used a large, multisite dataset and time-varying effect modeling (TVEM) to examine how ADHD symptom dimensions, including inattention and hyperactivity/impulsivity, relate to internalizing and externalizing problems across ages 3 to 18 in children and adolescents with NF1. We also tested whether these associations varied by sex, parental education, or NF1 inheritance mode. Our findings provide new insight into the potentially developmental presentation of ADHD-internalizing/externalizing comorbidity in NF1 and are discussed in detail below.

### 4.1. Robust Links Between ADHD Symptoms and Internalizing/Externalizing Problems Across Ages

Both inattention and hyperactivity/impulsivity were significantly associated with internalizing and externalizing problems among children and adolescents with NF1 from ages 3 to 17. In other words, higher levels of both ADHD symptom dimensions were linked to more severe internalizing or externalizing problems across childhood and adolescence in this population. These findings are consistent with findings in the general population [23], and highlight the comorbidity between ADHD symptoms and internalizing/externalizing problems in children and adolescents with NF1. One possible mechanism underlying these associations is executive function impairment, which has been linked to the development of both ADHD symptoms and internalizing and externalizing problems [55,56]. Children with NF1 are known to exhibit more pronounced executive function deficits than typically developing peers [4], which may place them at greater risk for a broad range of adjustment problems [6,7]. Future research should directly examine the role of executive function as a shared mechanism linking ADHD symptoms and comorbid emotional and behavioral difficulties to confirm whether executive dysfunction may be a transdiagnostic vulnerability factor in children and adolescents with NF1.

We also tested whether the strength of the associations varied across ages. The associations between inattention and both internalizing and externalizing problems as well as that between hyperactivity/impulsivity and internalizing problems were similar across ages, reflecting broad and enduring comorbidity between these problems across development. In contrast, we found a stronger association between hyperactivity/impulsivity and externalizing behaviors during childhood rather than adolescence. This result contrasts a finding in the general population where the correlation was stronger during adolescence [26], and thus, the finding may reflect a unique characteristic in NF1 population, which needs to be further tested and confirmed in future research. However, this finding is consistent with the general patterns of how self-regulation and behaviors develop or change developmentally. In young children, limited behavioral inhibition may manifest as impulsivity, aggression, or rule-breaking, which are core components of externalizing symptoms. The weakening of this association in adolescence likely reflects maturational improvements in self-regulation and increasing influence of other factors, such as peer influence, on behavior [14,15].

### 4.2. Moderating Role of NF1 Inheritance and the Robustness of Symptom Associations Across Demographic and Clinical Subgroups

Among the clinical and demographic moderators examined, only mode of NF1 inheritance significantly moderated associations between ADHD symptoms and the outcomes. Specifically, the link between inattention and externalizing problems was stronger from childhood to middle adolescence among children with familial NF1 compared to those with sporadic NF1. This finding extends prior work, which showed that children with NF1 whose parents also had NF1 experienced greater declines in several areas of neurocognitive functioning, including attention [34], by demonstrating that these children may also experience stronger links between attention problems and externalizing problems. It is possible that shared genetic liabilities or family-level environmental factors (e.g., parental neurocognitive difficulties, stress) contribute to more severe or multifaceted behavioral presentations in children with familial NF1, which should be examined in future research.

In contrast, no significant moderation effects emerged for sex or parental education. These null findings suggest that the associations between ADHD symptom dimensions and internalizing or externalizing problems are largely consistent across diverse demographic and clinical subgroups in children with NF1. The absence of moderation by sex or parental education is somewhat unexpected, given research indicating sex differences in ADHD comorbidities in the general population [31] as well as differences by parental education [32,33]. However, our results point to a degree of generalizability in these developmental associations within the NF1 population. Nonetheless, it remains possible that small interaction effects were not detected due to sample size limitations at certain ages. Future work using larger samples may help clarify whether subtle moderation patterns exist and how they manifest over time.

### 4.3. Implications

Although largely based on cross-sectional data, the results of this study offer several implications for clinical practice and research. First, the stronger association between hyperactivity/impulsivity and externalizing symptoms in early childhood highlights a key developmental window for intervention. Targeting behavioral regulation skills, such as impulse control and emotion management, during this stage may help reduce long-term behavioral risk. Second, the associations between inattention and hyperactivity/impulsivity and both internalizing and externalizing problems from early childhood through adolescence highlight ADHD symptom dimensions as potential correlates of broader emotional and behavioral challenges in NF1. These findings point to the value of considering ADHD symptoms alongside emotional and behavioral difficulties in assessment and treatment, and future research is needed to determine whether integrated intervention approaches should be used. Finally, the increased comorbidity observed in children with familial NF1 raises the possibility that parental NF1 status may be an important factor shaping child risk for co-occurring difficulties. Because checking family history of NF1 is already part of clinical practice, this finding has immediate relevance for identifying children who may be at elevated behavioral risk. Clinicians could consider closer monitoring, earlier screening, or more proactive support for behavioral and emotional difficulties in children with familial NF1. More broadly, these results underscore the need for future research to determine whether familial NF1 contributes to variation in developmental trajectories, treatment response, or service needs, which could inform the development of tailored interventions and early prevention strategies for high-risk subgroups.

### 4.4. Limitations and Future Directions

This study provides new insights into the potentially developmental patterns of ADHD-internalizing/externalizing comorbidities in NF1, but several limitations should be considered. First, the cross-sectional design limits inferences about intra-individual change as well as the directionality or causality of associations. While TVEM enables the estimation of age-varying associations, comparisons across age groups may be influenced by differences in comorbidity profiles, cognitive functioning, treatment exposure, or other unmeasured factors—issues that are particularly salient given the substantial clinical heterogeneity characteristic of NF1. Accordingly, longitudinal studies are needed to clarify causal mechanisms and determine whether the observed age-specific associations represent true developmental processes across childhood and adolescence.

Second, reliance on parent-report measures may introduce shared method variance and reporting bias. Future research should incorporate multi-informant approaches (e.g., teacher, self-report) and objective assessments of attention and behavior. Third, although measures were harmonized across sites using standardized scores, the use of multiple assessment instruments may have introduced residual measurement variability due to differences in scale content and psychometric properties. Such residual error may have attenuated effect estimates, particularly for interaction terms in moderation analyses. In addition, limited sample size at certain ages may have reduced statistical power to detect moderation effects. Future multisite studies employing consistent assessment measures and longitudinal designs will be critical for clarifying developmental trajectories and identifying meaningful subgroups.

Fourth, the internalizing composite included a somatization subscale, which may partly reflect genuine NF1-related physical symptoms (e.g., pain, headaches) rather than psychological distress. We retained somatization for construct consistency across instruments but acknowledge this potential overlap. Future studies should examine somatization separately to determine whether it reflects medical or psychological burden. Fifth, it is unclear whether these findings generalize to other rare disease populations. As NF1 has distinctive cognitive and behavioral characteristics, future research comparing children with other neurogenetic or chronic conditions could help identify which developmental patterns are specific to NF1 and which reflect broader adaptations to chronic illness, informing both targeted and cross-condition interventions. Finally, while familial NF1 emerged as a possible risk marker for greater inattention–externalizing comorbidity, underlying mechanisms remain unclear. Future research should investigate genetic, environmental, and neurobiological pathways, such as shared brain structure anomalies or intergenerational stress, to clarify how familial NF1 confers this risk. Taken together, given these limitations, the study findings should be considered exploratory and interpreted cautiously.

## 5. Conclusions

This study is among the first to examine associations between ADHD symptom dimensions and internalizing/externalizing problems across childhood and adolescence in NF1. Using a large, harmonized dataset and a developmentally sensitive modeling approach, we found that both inattention and hyperactivity/impulsivity were consistently linked to internalizing and externalizing difficulties across ages, with stronger impulsivity/hyperactivity–externalizing associations in childhood than in adolescence. These patterns were largely consistent across sex, parental education, and NF1 inheritance mode, although stronger inattention–externalizing comorbidity was observed in children with familial NF1. Overall, the findings highlight ADHD symptoms as transdiagnostic risk factors for broader emotional and behavioral problems, emphasizing the need for early identification and ongoing monitoring. From a translational perspective, interventions may be most effective when aligned with these developmental patterns. Early childhood interventions may prioritize behavioral regulation strategies targeting hyperactivity/impulsivity-related externalizing difficulties, whereas the persistence of both inattention- and hyperactivity/impulsivity-related associations across development highlights the need for sustained, developmentally appropriate attentional and self-regulatory supports. In adolescence, these supports may emphasize compensatory skill-building, executive function strategies, self-monitoring, and transition-focused planning to promote academic and social functioning. Interventions that also address family-level risk factors, such as parental NF1, may further support adaptive outcomes. Finally, future multi-site longitudinal studies are needed to clarify causal pathways and refine the optimal timing and integration of interventions across development.

## Figures and Tables

**Table 1:** Sample Sizes for the Associations between ADHD Symptoms and Internalizing Problems across Ages.

Predictor(s) in Each Model	Age Group (in Years)															
	3.00–3.99	4.00–4.99	5.00–5.99	6.00–6.99	7.00–7.99	8.00–7.99	9.00–9.99	10.00–10.99	11.00–11.99	12.00–12.99	13.00–13.99	14.00–14.99	15.00–15.99	16.00–16.99	17.00–17.99	18.00–18.99
Inattention	38	41	48	62	59	53	69	59	47	49	44	31	29	25	22	6
Inattention + sex + inattention × sex	38	41	47	61	59	53	68	59	47	49	43	31	29	25	22	6
Inattention by sex																
Male	25	24	29	34	25	28	44	33	22	34	24	17	16	15	14	3
Female	13	17	18	27	34	25	24	26	25	15	19	14	13	10	8	3
Inattention + parental education + inattention × parental education	35	40	40	50	44	37	50	47	39	42	35	26	24	20	20	1
Inattention by parental education																
Lower than college	10	7	13	17	14	9	11	14	15	14	13	13	11	8	8	0
Some college or above	25	33	27	33	30	28	39	33	24	28	22	13	13	12	12	1
Inattention + NF1 inheritance + inattention × NF1 inheritance	35	41	41	51	46	41	53	47	38	44	34	26	24	22	19	1
Inattention by NF1 inheritance																
Sporadic NF1	20	27	27	26	34	27	37	28	22	26	22	14	14	13	11	1
Familial NF1	15	14	14	25	12	14	16	19	16	18	12	12	10	9	8	0
Hyperactivity/impulsivity	38	41	49	62	59	55	69	59	47	49	43	31	29	25	22	6
Hyperactivity/impulsivity + sex + hyperactivity/impulsivity × sex	38	41	48	61	59	55	68	59	47	49	42	31	29	25	22	6
Hyperactivity/impulsivity by sex																
Male	25	24	29	34	25	29	44	33	22	34	24	17	16	15	14	3
Female	13	17	19	27	34	26	24	26	25	15	18	14	13	10	8	3
Hyperactivity/impulsivity + parental education + hyperactivity/impulsivity × parental education	35	40	41	50	44	39	50	47	39	42	35	26	24	20	20	1
Hyperactivity/impulsivity by parental education																
Lower than college	10	7	13	17	14	10	11	14	15	14	13	13	11	8	8	0
Some college or above	25	33	28	33	30	29	39	33	24	28	22	13	13	12	12	1
Hyperactivity/impulsivity + NF1 inheritance + hyperactivity/impulsivity × NF1 inheritance	35	41	41	51	46	43	53	47	38	44	34	26	24	22	19	1
Hyperactivity/impulsivity by NF1 inheritance																
Sporadic NF1	20	27	27	26	34	28	37	28	22	26	22	14	14	13	11	1
Familial NF1	15	14	14	25	12	15	16	19	16	18	12	12	10	9	8	0

*Note*. ADHD = attention deficit/hyperactivity disorder. NF1 = neurofibromatosis type 1.

**Table 2:** Sample Sizes for the Associations between ADHD Symptoms and Externalizing Problems across Ages.

Predictor(s) in Each Model	Age Group (in Years)															
	3.00–3.99	4.00–4.99	5.00–5.99	6.00–6.99	7.00–7.99	8.00–7.99	9.00–9.99	10.00–10.99	11.00–11.99	12.00–12.99	13.00–13.99	14.00–14.99	15.00–15.99	16.00–16.99	17.00–17.99	18.00–18.99
Inattention	38	41	48	62	59	53	69	59	47	49	44	31	29	25	22	6
Inattention + sex + inattention × sex	38	41	47	61	59	53	68	59	47	49	43	31	29	25	22	6
Inattention by sex																
Male	25	24	29	34	25	28	44	33	22	34	24	17	16	15	14	3
Female	13	17	18	27	34	25	24	26	25	15	19	14	13	10	8	3
Inattention + parental education + inattention × parental education	35	40	40	50	44	37	50	47	39	42	35	26	24	20	20	1
Inattention by parental education																
Lower than college	10	7	13	17	14	9	11	14	15	14	13	13	11	8	8	0
Some college or above	25	33	27	33	30	28	39	33	24	28	22	13	13	12	12	1
Inattention + NF1 inheritance + inattention × NF1 inheritance	35	41	41	51	46	41	53	47	38	44	34	26	24	22	19	1
Inattention by NF1 inheritance																
Sporadic NF1	20	27	27	26	34	27	37	28	22	26	22	14	14	13	11	1
Familial NF1	15	14	14	25	12	14	16	19	16	18	12	12	10	9	8	0
Hyperactivity/impulsivity	38	41	49	62	59	55	69	59	47	49	43	31	29	25	22	6
Hyperactivity/impulsivity + sex + hyperactivity/impulsivity × sex	38	41	48	61	59	55	68	59	47	49	42	31	29	25	22	6
Hyperactivity/impulsivity by sex																
Male	25	24	29	34	25	29	44	33	22	34	24	17	16	15	14	3
Female	13	17	19	27	34	26	24	26	25	15	18	14	13	10	8	3
Hyperactivity/impulsivity + parental education + hyperactivity/impulsivity × parental education	35	40	41	50	44	39	50	47	39	42	35	26	24	20	20	1
Hyperactivity/impulsivity by parental education																
Lower than college	10	7	13	17	14	10	11	14	15	14	13	13	11	8	8	0
Some college or above	25	33	28	33	30	29	39	33	24	28	22	13	13	12	12	1
Hyperactivity/impulsivity + NF1 inheritance + hyperactivity/impulsivity × NF1 inheritance	35	41	41	51	46	43	53	47	38	44	34	26	24	22	19	1
Hyperactivity/impulsivity by NF1 inheritance																
Sporadic NF1	20	27	27	26	34	28	37	28	22	26	22	14	14	13	11	1
Familial NF1	15	14	14	25	12	15	16	19	16	18	12	12	10	9	8	0

*Note*. ADHD = attention deficit/hyperactivity disorder. NF1 = neurofibromatosis type 1.

## Data Availability

Main study data are included in the manuscript and Supplementary Materials. Additional data are available upon request sent to the corresponding author.

## Supplementary Materials

The following supporting information can be downloaded at: https://www.mdpi.com/article/10.3390/cancers18030529/s1. Supplementary Materials include the following content: Supplementary Descriptive Statistics; Table S1. Characteristics of Data for Each Site and the Combined Sample; Table S2. Demographic, NF1 Disease-Related, and Clinical Characteristics Assessed at Each Site and in the Combined Sample; Table S3. Sample Size Distribution by Age for Participants Assessed with CBCL and BASC; Table S4. Descriptives and Correlations of Study Variables; Table S5. TVEM Estimates for Simple Associations between ADHD Symptoms and Internalizing Problems across Ages; Table S6. TVEM Estimates for Simple Associations between ADHD Symptoms and Externalizing Problems across Ages; Table S7. Model Fit Comparison Across Random Effect Specifications; Figure S1. Age-Varying Associations between ADHD Symptoms and Internalizing Problems Moderated by Sex; Figure S2. Age-Varying Associations between ADHD Symptoms and Internalizing Problems Moderated by Parental Education; Figure S3. Age-Varying Associations between ADHD Symptoms and Internalizing Problems Moderated by Mode of NF1 Inheritance; Figure S4. Age-Varying Associations between ADHD Symptoms and Externalizing Problems Moderated by Sex; Figure S5. Age-Varying Associations between ADHD Symptoms and Externalizing Problems Moderated by Parental Education; Figure S6. Age-Varying Associations between ADHD Symptoms and Externalizing Problems Moderated by Mode of NF1 Inheritance; Figure S7. Age-Varying Associations between ADHD Symptoms and Internalizing Problems Controlling for CBCL or BASC Instrument Type; Figure S8. Age-Varying Associations between ADHD Symptoms and Externalizing Problems Controlling for CBCL or BASC Instrument Type.

## Abbreviations

ADHD: attention-deficit/hyperactivity disorder; ADHD RS-IV: ADHD Rating Scale-IV: Home Version; BASC: Behavior Assessment System for Children; CBCL: Child Behavior Checklist; CI: confidence interval; Conners 3-P: Conners-3 Parent Report; CRS: Conners Rating Scales; ID: identification; IDA: integrative data analysis; IRB: institutional review board; NF1: neurofibromatosis type 1; PNs: plexiform neurofibromas; Ras/MAPK: the Ras/Mitogen-Activated Protein Kinase signaling pathway; SD: standard deviation; TVEM: time-varying effect modeling.
