# Supplementary material for: Age-Specific ADHD and Internalizing/Externalizing Comorbidity in Children with Neurofibromatosis Type 1: A Multi-Site Study
Source: Cancers (Basel). 2026 Feb 6;18(3):529. doi: 10.3390/cancers18030529 (PMC12897463; doi:10.3390/cancers18030529)
Supplement: Supplementary file 1 [file cancers-18-00529-s001.zip › cancers-4048895-supplementary.pdf]

# Supplementary Information

## Age-Specific ADHD and Internalizing/Externalizing Comorbidity in Children with Neurofibromatosis Type 1: A Multi-Site Study

*Cancers*

Dan Liu [1], Pamela L. Wolters [2], Bonita P. Klein-Tasman [3], Karin S. Walsh [4,5], Jonathan M. Payne [6,7], Natalie Pride [8], Stephanie M. Morris [9] and Yang Hou [1,*]

1 Department of Behavioral Sciences and Social Medicine, College of Medicine, Florida State University, Tallahassee, FL 32306, USA; dan.liu@med.fsu.edu
2 Pediatric Oncology Branch, Center for Cancer Research, National Cancer Institute, National Institutes of Health, Bethesda, MD 20892, USA; woltersp@mail.nih.gov
3 Department of Psychology, University of Wisconsin–Milwaukee, Milwaukee, WI 53211, USA; bklein@uwm.edu
4 Children's National Hospital, Washington, DC 20010, USA; kwalsh@childrensnational.org
5 Department of Psychiatry & Behavioral Health, George Washington University School of Medicine, Washington, DC 20037, USA
6 Murdoch Children's Research Institute, Melbourne 3052, Australia; jonathan.payne@mcri.edu.au
7 Department of Paediatrics, University of Melbourne, Melbourne 3010, Australia
8 Children's Hospital atWestmead, Sydney 2145, Australia; natalie.pride@health.nsw.gov.au
9 Kennedy Krieger Institute, Baltimore, MD 21211, USA; morrisst@kennedykrieger.org
* Correspondence: yang.hou@med.fsu.edu

## Table of Contents

**Supplementary Descriptive Statistics**........3
Table S1. Characteristics of Data for Each Site and the Combined Sample........4
Table S2. Demographic, NF1 Disease-Related, and Clinical Characteristics Assessed at Each Site and in the Combined Sample........5
Table S3. Sample Size Distribution by Age for Participants Assessed with CBCL and BASC........7
Table S4. Descriptives and Correlations of Study Variables........8
Table S5. TVEM Estimates for Simple Associations between ADHD Symptoms and Internalizing Problems across Ages .....9
Table S6. TVEM Estimates for Simple Associations between ADHD Symptoms and Externalizing Problems across Ages 122
Table S7. Model Fit Comparison Across Random Effect Specifications........155
Figure S1. Age-Varying Associations between ADHD Symptoms and Internalizing Problems Moderated by Sex........17
Figure S2. Age-Varying Associations between ADHD Symptoms and Internalizing Problems Moderated by Parental Education........18
Figure S3. Age-Varying Associations between ADHD Symptoms and Internalizing Problems Moderated by Mode of NF1 Inheritance........19
Figure S4. Age-Varying Associations between ADHD Symptoms and Externalizing Problems Moderated by Sex ........200
Figure S5. Age-Varying Associations between ADHD Symptoms and Externalizing Problems Moderated by Parental Education........211
Figure S6. Age-Varying Associations between ADHD Symptoms and Externalizing Problems Moderated by Mode of NF1 Inheritance........22
Figure S7. Age-Varying Associations between ADHD Symptoms and Internalizing Problems Controlling for CBCL or BASC Instrument Type........23
Figure S8. Age-Varying Associations between ADHD Symptoms and Externalizing Problems Controlling for CBCL or BASC Instrument Type........24

## Supplementary Descriptive Statistics

Table S4 presents the descriptives and correlations of all study variables. The means of the ADHD variables as well as internalizing and externalizing problems in children with NF1 were higher than the normative mean of 50. Inattention and hyperactivity/impulsivity were positively and strongly correlated. Each ADHD variable was also moderately to strongly correlated with internalizing and externalizing problems, with all correlations being positive. Compared with younger children with NF1, the gaps between older children with NF1 and the normative sample were wider in internalizing problems but narrower in externalizing problems. Females showed higher levels of inattention symptoms, hyperactivity/impulsivity symptoms, and internalizing problems than males. Children with higher parental education showed lower levels of inattention symptoms, hyperactivity/impulsivity symptoms, internalizing problems, and externalizing problems, compared with those with lower parental education. Children with familial NF1 exhibited higher levels of hyperactivity/impulsivity and externalizing problems, compared with those with sporadic NF1.

Table S1. Characteristics of Data for Each Site and the Combined Sample

| **Characteristic** | **National Cancer Institute, USA** | **University of Wisconsin–Milwaukee, USA** | **Children's National Hospital, USA** | **Murdoch Children's Research Institute, Australia** | **Children's Hospital at Westmead, Australia** | **Washington University in St. Louis, USA** | **Combined** |
|---|---|---|---|---|---|---|---|
| Recruitment population | Convenience sample enrolled in a natural history study recruited nationally (clinicaltrial.gov number: NCT00924196) | Convenience sample from NF1 clinic | Convenience sample recruited through patients seen for clinical evaluation in a pre-clinical appointment survey from a pediatric neuropsychology clinic | Convenience sample recruited on a sequential basis from a multidisciplinary NF1 Clinic | Convenience sample recruited on a sequential basis from a neurogenetics clinic | Convenience sample from NF1 outpatient clinic | NA |
| Measure of Internalizing Problems | BASC 2 | BASC 2 | CBCL/1.5-5, 6-18 | CBCL/6-18 | CBCL/1.5-5, 6-18 | BASC 2 | NA |
| Measure of Externalizing Problems | BASC 2 | BASC 2 | CBCL/1.5-5, 6-18 | CBCL/6-18 | CBCL/1.5-5, 6-18 | BASC 2 | NA |
| Measure of ADHD Symptoms | CPRS-R:S | Conners 3 (S); CPRS-R | CADS; Conners 3; ADHD RS IV | CADS; Conners 3 | CADS; Conners 3 | Conners 3 | |
| Total *N.*[a] | 266 | 155 | 89 | 72 | 60 | 43 | 685 |
| Internalizing *N.* | 266 | 155 | 89 | 72 | 60 | 43 | 685 |
| Externalizing *N.* | 266 | 155 | 89 | 72 | 60 | 43 | 685 |
| Inattention *N.* | 265 | 155 | 89 | 71 | 60 | 42 | 682 |
| Hyperactivity/Impulsivity *N.* | 266 | 155 | 89 | 72 | 59 | 43 | 684 |
| Age, median (range), y | 11.3 (3-18) | 6.3 (3-13) | 9.5 (3-18) | 10.1 (6-15) | 10.0 (3-16) | 9.8 (6-16) | 9.5 (3-18) |
| Sex, *N.* | 266 | 155 | 86 | 71 | 60 | 43 | 681 |
| Male, *N.* (%) | 157 (59) | 94 (61) | 44 (51) | 41 (58) | 30 (50) | 22 (51) | 388 (57) |
| Female, *N.* (%) | 109 (41) | 61 (39) | 42 (49) | 30 (42) | 30 (50) | 21 (49) | 293(43) |
| Parent education, *N.* | 256 | 155 | 7 | 70 | 39 | 26 | 553 |
| Low,[b] *N.* (%) | 89 (35) | 37 (24) | 0 (0) | 29 (41) | 11 (28) | 12 (46) | 178 (32) |
| High,[b] *N.* (%) | 167 (65) | 118 (76) | 7 (100) | 41 (59) | 28 (72) | 14 (54) | 375 (68) |
| NF1 heritability, *N.* | 246 | 153 | 0 | 72 | 51 | 43 | 565 |
| Sporadic, *N.* (%) | 150 (61) | 100 (65) | 0 | 48 (67) | 30 (59) | 22 (51) | 350 (62) |
| Familial, *N.* (%) | 96 (39) | 53 (35) | 0 | 24 (33) | 21 (41) | 21 (49) | 215 (38) |
| Plexiform neurofibromas, *N* | 218 | 38 | 0 | 51 | 18 | 0 | 325 |
| No, *N* (%) | 21 (10) | 26 (68) | 0 | 39 (76) | 14 (78) | 0 | 100 (31) |
| Yes, *N* (%) | 197 (90) | 12 (32) | 0 | 12 (24) | 4 (22) | 0 | 225 (69) |

*Note.* N. = valid number of observations. y = age in years. NF1 = neurofibromatosis type 1. NA = not applicable. BASC = Behavior Assessment System for Children. CBCL = Child Behavior Checklist. CPRS-R:S = Conners Rating Scales – Revised Short. Conners 3 (S) = Conners Rating Scales, version 3-Short Form. CPRS-R = Conners Rating Scales – Revised. CADS = Conners ADHD/DSM-IV Scales. Conners 3 = Conners Rating Scales, 3rd version. ADHD RS IV = ADHD Rating Scales IV. [a]The reported sample sizes are based on available data encompassing both ADHD and internalizing or externalizing variables. [b]Low = high school or lower education; High = some college or higher education.

Table S2. Demographic, NF1 Disease-Related, and Clinical Characteristics Assessed at Each Site and in the Combined Sample

| **Characteristics** | **National Cancer Institute, USA** | **University of Wisconsin–Milwaukee, USA** | **Children's National Hospital, USA** | **Murdoch Children's Research Institute, Australia** | **Children's Hospital at Westmead, Australia** | **Washington University in St. Louis, USA** | **Combined** |
|---|---|---|---|---|---|---|---|
| Informant | Demographic information and NF1 Heritability were reported by parents or primary caregivers; other variables were rated by medical professionals | Reported by parents or primary caregivers | Demographic information was reported by parents or primary caregivers; NF1-related disease characteristics were rated by medical professionals | Reported by parents or primary caregivers | Reported by parents or primary caregivers | Medical records | NA |
| Child sex | 0 = Female, 1 = Male | 1 = Male, 2 = Female | 1 = Male, 2 = Female, 3 = Gender nonconforming or transgender | 1 = Male, 2 = Female | 1 = Male, 2 = Female | 0 = Male, 1= Female | 0 = Male, 1= Female |
| Parental education | Mother's/ Father's number of years of education | Highest maternal/ paternal education: 1 = Less than 7th grade, 2 = 8th or 9th grade, 3 = 10th or 11th grade, 4 = High school graduate/ G.E.D., 5 = Partial college, 6 = College/University graduate, 7 = Graduate professional training | Maternal caregiver's highest level of education: 1 = 6 years or less, 2 = 7-9 years, 3 = 10-11 years, 4 = at least 12 years, 5 = some college or university, 6 = college or university graduate, 7 = post-graduate training | Maternal/Paternal caregiver's highest level of education: 1 = 6 years or less, 2 = 7-9 years, 3 = 10-11 years, 4 = at least 12 years, 5 = some college or university, 6 = college or university graduate, 7 = post-graduate training | Maternal/Paternal caregiver's highest level of education: 1 = 6 years or less, 2 = 7-9 years, 3 = 10-11 years, 4 = at least 12 years, 5 = some college or university, 6 = college or university graduate, 7 = post-graduate training | Highest maternal/paternal education: 0 = Less than 12 years, 1 = High school graduate/G.E.D., 2 = Some college/associate degree, 3 = Standard college/university graduate, 4 = Master's degree, 5 = Doctoral degree | 0 = Low (lower than college), 1 = High (some college or above) |
| NF1 inheritance | NF1 status of father/mother: 1 = Affected, 2 = No | 1 = Familial, 2 = Sporadic | NA | 1 = Sporadic, 2 = Familial | 1 = Sporadic, 2 = Familial | Family History of NF1: 0 = No, 1 = Yes | 0 = Sporadic (neither parent had |

| Characteristics | National Cancer Institute, USA | University of Wisconsin–Milwaukee, USA | Children’s National Hospital, USA | Murdoch Children's Research Institute, Australia | Children's Hospital at Westmead, Australia | Washington University in St. Louis, USA | Combined |
|---|---|---|---|---|---|---|---|
| | | | | | | | NF1), 1 = Familial (at least one parent had NF1) |
| Plexiform neurofibromas | 0 = Absent, 1 = Present | TRUE = Yes, FALSE = No | NA | 1 = Yes, 2 = No | 1 = Yes, 2 = No | NA | 0 = no; 1 = yes |

*Note*. NF1 = neurofibromatosis type 1. NA = not applicable.

Table S3. Sample Size Distribution by Age for Participants Assessed with CBCL and BASC

| **Measure** | **Age Group (in years)** | | | | | | | | | | | | | | | |
|---|---|---|---|---|---|---|---|---|---|---|---|---|---|---|---|---|
| | **3.00-3.99** | **4.00-4.99** | **5.00-5.99** | **6.00-6.99** | **7.00-7.99** | **8.00-7.99** | **9.00-9.99** | **10.00-10.99** | **11.00-11.99** | **12.00-12.99** | **13.00-13.99** | **14.00-14.99** | **15.00-15.99** | **16.00-16.99** | **17.00-17.99** | **18.00-18.99** |
| CBCL | 36 | 41 | 42 | 40 | 31 | 33 | 39 | 39 | 24 | 33 | 33 | 22 | 20 | 21 | 21 | 1 |
| BASC | 2 | 0 | 7 | 22 | 28 | 22 | 30 | 20 | 23 | 16 | 11 | 9 | 9 | 4 | 1 | 5 |

*Note*. CBCL = Child Behavior Checklist. BASC = Behavior Assessment System for Children.

Table S4. Descriptives and Correlations of Study Variables

| | 1 | 2 | 3 | 4 | 5 | 6 | 7 | 8 |
|---|---|---|---|---|---|---|---|---|
| 1. Inattention | | | | | | | | |
| 2. Hyperactivity/impulsivity | 0.69*** | | | | | | | |
| 3. Internalizing problems | 0.34*** | 0.34*** | | | | | | |
| 4. Externalizing problems | 0.50*** | 0.62*** | 0.47*** | | | | | |
| 5. Age | -0.02 | -0.05 | 0.11** | -0.12** | | | | |
| 6. Sex[1] | 0.11** | 0.13** | 0.10* | 0.04 | 0.00 | | | |
| 7. Parental education[2] | -0.10* | -0.22*** | -0.15*** | -0.12** | -0.16*** | -0.06 | | |
| 8. Mode of NF1 Inheritance[3] | 0.03 | 0.11** | 0.03 | 0.13** | 0.03 | -0.05 | -0.38*** | |
| Total *n* | 682 | 684 | 685 | 685 | 685 | 681 | 553 | 565 |
| Mean/*n*[4] | 62.66 | 61.10 | 55.68 | 53.06 | 9.79 | 293 | 3.29 | 215 |
| *SD*/%[5] | 14.76 | 15.50 | 11.95 | 11.14 | 3.88 | 43.0 | 0.98 | 38.1 |
| % missingness | 0.44 | 0.15 | 0.00 | 0.00 | 0.00 | 0.58 | 19.27 | 17.52 |

*Note*. NF1 = neurofibromatosis type 1. n = valid number of observations. SD = standard deviation. [1]male = 0, female = 1; [2]less than high school = 1, high school or partial high school = 2, some college, community college, or associate degree = 3, college or university degree = 4, graduate or professional training = 5; [3]sporadic NF1 = 0, familial NF1 =1; [4]Mean for continuous variables, and sample size for the indicator of dichotomized variables. [5]Standard deviation for continuous variables, and percentage for the indicator of dichotomized variables.

**p* < .05. ***p* < .01. ****p* < .001.

Table S5. TVEM Estimates for Simple Associations between ADHD Symptoms and Internalizing Problems across Ages

| | **Inattention-Internalizing** | | | **Hyperactivity/Impulsivity-Internalizing** | | |
|---|---|---|---|---|---|---|
| **Age** | **95% CI_LL** | **Intercept** | **95% CI_UL** | **95% CI_LL** | **Intercept** | **95% CI_UL** |
| 3.00 | 0.00 | 0.33 | 0.65 | 0.06 | 0.32 | 0.57 |
| 3.16 | 0.03 | 0.33 | 0.63 | 0.08 | 0.32 | 0.56 |
| 3.32 | 0.06 | 0.33 | 0.61 | 0.10 | 0.32 | 0.54 |
| 3.48 | 0.08 | 0.34 | 0.59 | 0.12 | 0.33 | 0.53 |
| 3.64 | 0.10 | 0.34 | 0.58 | 0.14 | 0.33 | 0.52 |
| 3.80 | 0.12 | 0.34 | 0.56 | 0.16 | 0.33 | 0.51 |
| 3.96 | 0.14 | 0.34 | 0.55 | 0.17 | 0.34 | 0.50 |
| 4.12 | 0.15 | 0.34 | 0.53 | 0.18 | 0.34 | 0.49 |
| 4.28 | 0.17 | 0.34 | 0.52 | 0.20 | 0.34 | 0.48 |
| 4.44 | 0.18 | 0.34 | 0.51 | 0.20 | 0.34 | 0.47 |
| 4.60 | 0.19 | 0.34 | 0.49 | 0.21 | 0.34 | 0.47 |
| 4.76 | 0.20 | 0.34 | 0.48 | 0.22 | 0.34 | 0.46 |
| 4.92 | 0.21 | 0.34 | 0.48 | 0.22 | 0.34 | 0.45 |
| 5.08 | 0.21 | 0.34 | 0.47 | 0.23 | 0.34 | 0.45 |
| 5.24 | 0.22 | 0.34 | 0.46 | 0.23 | 0.34 | 0.44 |
| 5.40 | 0.22 | 0.34 | 0.46 | 0.23 | 0.34 | 0.44 |
| 5.56 | 0.22 | 0.34 | 0.45 | 0.23 | 0.33 | 0.43 |
| 5.72 | 0.23 | 0.34 | 0.45 | 0.23 | 0.33 | 0.43 |
| 5.88 | 0.23 | 0.33 | 0.44 | 0.23 | 0.33 | 0.42 |
| 6.04 | 0.23 | 0.33 | 0.44 | 0.23 | 0.33 | 0.42 |
| 6.20 | 0.22 | 0.33 | 0.43 | 0.23 | 0.32 | 0.42 |
| 6.36 | 0.22 | 0.33 | 0.43 | 0.23 | 0.32 | 0.41 |
| 6.52 | 0.22 | 0.32 | 0.43 | 0.22 | 0.32 | 0.41 |
| 6.68 | 0.22 | 0.32 | 0.42 | 0.22 | 0.31 | 0.40 |
| 6.84 | 0.21 | 0.32 | 0.42 | 0.22 | 0.31 | 0.40 |
| 7.00 | 0.21 | 0.32 | 0.42 | 0.22 | 0.31 | 0.40 |
| 7.16 | 0.21 | 0.31 | 0.42 | 0.21 | 0.30 | 0.39 |
| 7.32 | 0.21 | 0.31 | 0.41 | 0.21 | 0.30 | 0.39 |
| 7.48 | 0.20 | 0.31 | 0.41 | 0.20 | 0.29 | 0.38 |
| 7.64 | 0.20 | 0.30 | 0.40 | 0.20 | 0.29 | 0.38 |
| 7.80 | 0.20 | 0.30 | 0.40 | 0.20 | 0.28 | 0.37 |
| 7.96 | 0.20 | 0.30 | 0.40 | 0.19 | 0.28 | 0.37 |
| 8.12 | 0.19 | 0.29 | 0.39 | 0.19 | 0.27 | 0.36 |
| 8.28 | 0.19 | 0.29 | 0.39 | 0.19 | 0.27 | 0.35 |
| 8.44 | 0.19 | 0.28 | 0.38 | 0.18 | 0.27 | 0.35 |
| 8.60 | 0.19 | 0.28 | 0.38 | 0.18 | 0.26 | 0.34 |
| 8.76 | 0.18 | 0.28 | 0.37 | 0.17 | 0.26 | 0.34 |

| Age | Inattention-Internalizing | | | Hyperactivity/Impulsivity-Internalizing | | |
|---|---|---|---|---|---|---|
| | 95% CI_LL | Intercept | 95% CI_UL | 95% CI_LL | Intercept | 95% CI_UL |
| 8.92 | 0.18 | 0.27 | 0.37 | 0.17 | 0.25 | 0.33 |
| 9.08 | 0.18 | 0.27 | 0.36 | 0.17 | 0.25 | 0.33 |
| 9.24 | 0.18 | 0.27 | 0.36 | 0.16 | 0.24 | 0.32 |
| 9.40 | 0.17 | 0.26 | 0.36 | 0.16 | 0.24 | 0.32 |
| 9.56 | 0.17 | 0.26 | 0.35 | 0.16 | 0.23 | 0.31 |
| 9.72 | 0.17 | 0.26 | 0.35 | 0.15 | 0.23 | 0.31 |
| 9.88 | 0.16 | 0.25 | 0.34 | 0.15 | 0.22 | 0.30 |
| 10.04 | 0.16 | 0.25 | 0.34 | 0.14 | 0.22 | 0.30 |
| 10.20 | 0.16 | 0.25 | 0.34 | 0.14 | 0.22 | 0.29 |
| 10.36 | 0.16 | 0.25 | 0.34 | 0.14 | 0.21 | 0.29 |
| 10.52 | 0.15 | 0.24 | 0.33 | 0.13 | 0.21 | 0.29 |
| 10.68 | 0.15 | 0.24 | 0.33 | 0.13 | 0.21 | 0.29 |
| 10.84 | 0.15 | 0.24 | 0.33 | 0.12 | 0.20 | 0.28 |
| 11.00 | 0.14 | 0.24 | 0.33 | 0.12 | 0.20 | 0.28 |
| 11.16 | 0.14 | 0.23 | 0.33 | 0.12 | 0.20 | 0.28 |
| 11.32 | 0.14 | 0.23 | 0.33 | 0.11 | 0.20 | 0.28 |
| 11.48 | 0.13 | 0.23 | 0.33 | 0.11 | 0.19 | 0.28 |
| 11.64 | 0.13 | 0.23 | 0.33 | 0.11 | 0.19 | 0.28 |
| 11.80 | 0.13 | 0.23 | 0.33 | 0.10 | 0.19 | 0.28 |
| 11.96 | 0.12 | 0.23 | 0.33 | 0.10 | 0.19 | 0.28 |
| 12.12 | 0.12 | 0.23 | 0.33 | 0.10 | 0.19 | 0.28 |
| 12.28 | 0.12 | 0.23 | 0.33 | 0.10 | 0.19 | 0.29 |
| 12.44 | 0.12 | 0.23 | 0.34 | 0.09 | 0.19 | 0.29 |
| 12.60 | 0.11 | 0.23 | 0.34 | 0.09 | 0.19 | 0.29 |
| 12.76 | 0.11 | 0.23 | 0.34 | 0.09 | 0.19 | 0.29 |
| 12.92 | 0.11 | 0.23 | 0.34 | 0.09 | 0.19 | 0.30 |
| 13.08 | 0.11 | 0.23 | 0.35 | 0.09 | 0.20 | 0.30 |
| 13.24 | 0.11 | 0.23 | 0.35 | 0.09 | 0.20 | 0.31 |
| 13.40 | 0.11 | 0.23 | 0.35 | 0.09 | 0.20 | 0.31 |
| 13.56 | 0.11 | 0.23 | 0.36 | 0.10 | 0.21 | 0.32 |
| 13.72 | 0.11 | 0.24 | 0.36 | 0.10 | 0.21 | 0.32 |
| 13.88 | 0.12 | 0.24 | 0.36 | 0.10 | 0.22 | 0.33 |
| 14.04 | 0.12 | 0.24 | 0.37 | 0.10 | 0.22 | 0.34 |
| 14.20 | 0.12 | 0.25 | 0.37 | 0.11 | 0.23 | 0.34 |
| 14.36 | 0.12 | 0.25 | 0.38 | 0.11 | 0.23 | 0.35 |
| 14.52 | 0.13 | 0.26 | 0.38 | 0.12 | 0.24 | 0.36 |
| 14.68 | 0.13 | 0.26 | 0.39 | 0.12 | 0.25 | 0.37 |
| 14.84 | 0.13 | 0.27 | 0.40 | 0.13 | 0.26 | 0.38 |

| Age | Inattention-Internalizing | | | Hyperactivity/Impulsivity-Internalizing | | |
|---|---|---|---|---|---|---|
| | 95% CI_LL | Intercept | 95% CI_UL | 95% CI_LL | Intercept | 95% CI_UL |
| 15.00 | 0.14 | 0.27 | 0.41 | 0.14 | 0.27 | 0.40 |
| 15.16 | 0.14 | 0.28 | 0.41 | 0.14 | 0.28 | 0.41 |
| 15.32 | 0.15 | 0.29 | 0.42 | 0.15 | 0.29 | 0.42 |
| 15.48 | 0.15 | 0.29 | 0.43 | 0.16 | 0.30 | 0.44 |
| 15.64 | 0.16 | 0.30 | 0.45 | 0.16 | 0.31 | 0.45 |
| 15.80 | 0.16 | 0.31 | 0.46 | 0.17 | 0.32 | 0.47 |
| 15.96 | 0.16 | 0.32 | 0.47 | 0.18 | 0.34 | 0.49 |
| 16.12 | 0.17 | 0.33 | 0.49 | 0.19 | 0.35 | 0.51 |
| 16.28 | 0.17 | 0.34 | 0.51 | 0.19 | 0.36 | 0.54 |
| 16.44 | 0.17 | 0.35 | 0.53 | 0.20 | 0.38 | 0.56 |
| 16.60 | 0.17 | 0.36 | 0.55 | 0.21 | 0.40 | 0.59 |
| 16.76 | 0.17 | 0.37 | 0.57 | 0.22 | 0.41 | 0.61 |
| 16.92 | 0.17 | 0.38 | 0.60 | 0.22 | 0.43 | 0.64 |
| 17.08 | 0.17 | 0.40 | 0.62 | 0.23 | 0.45 | 0.68 |
| 17.24 | 0.17 | 0.41 | 0.65 | 0.24 | 0.47 | 0.71 |
| 17.40 | 0.17 | 0.43 | 0.68 | 0.24 | 0.49 | 0.75 |
| 17.56 | 0.16 | 0.44 | 0.72 | 0.25 | 0.52 | 0.78 |
| 17.72 | 0.16 | 0.46 | 0.75 | 0.26 | 0.54 | 0.82 |
| 17.88 | 0.15 | 0.47 | 0.79 | 0.26 | 0.56 | 0.86 |
| 18.04 | 0.15 | 0.49 | 0.83 | 0.27 | 0.59 | 0.91 |
| 18.20 | 0.14 | 0.51 | 0.87 | 0.27 | 0.61 | 0.95 |
| 18.36 | 0.13 | 0.53 | 0.92 | 0.28 | 0.64 | 1.00 |
| 18.52 | 0.13 | 0.54 | 0.96 | 0.29 | 0.67 | 1.05 |
| 18.68 | 0.12 | 0.56 | 1.01 | 0.29 | 0.70 | 1.11 |
| 18.84 | 0.11 | 0.59 | 1.06 | 0.30 | 0.73 | 1.16 |

*Note.* TVEM = time-varying effect modeling. ADHD = attention deficit/hyperactivity disorder. CI = confidence interval. LL = lower limit. UL = upper limit.

Table S6. TVEM Estimates for Simple Associations between ADHD Symptoms and Externalizing Problems across Ages

| | Inattention-Externalizing | | | Hyperactivity/Impulsivity-Externalizing | | |
|---|---|---|---|---|---|---|
| Age | 95% CI_LL | Intercept | 95% CI_UL | 95% CI_LL | Intercept | 95% CI_UL |
| 3.00 | -0.10 | 0.21 | 0.52 | 0.21 | 0.44 | 0.66 |
| 3.16 | -0.05 | 0.23 | 0.52 | 0.25 | 0.45 | 0.66 |
| 3.32 | -0.01 | 0.25 | 0.52 | 0.28 | 0.47 | 0.66 |
| 3.48 | 0.03 | 0.27 | 0.51 | 0.30 | 0.48 | 0.65 |
| 3.64 | 0.07 | 0.29 | 0.51 | 0.33 | 0.49 | 0.65 |
| 3.80 | 0.10 | 0.31 | 0.51 | 0.35 | 0.50 | 0.65 |
| 3.96 | 0.13 | 0.32 | 0.51 | 0.37 | 0.51 | 0.65 |
| 4.12 | 0.16 | 0.34 | 0.51 | 0.39 | 0.52 | 0.65 |
| 4.28 | 0.19 | 0.35 | 0.51 | 0.41 | 0.53 | 0.65 |
| 4.44 | 0.22 | 0.36 | 0.51 | 0.42 | 0.54 | 0.65 |
| 4.60 | 0.24 | 0.37 | 0.51 | 0.44 | 0.54 | 0.65 |
| 4.76 | 0.26 | 0.38 | 0.51 | 0.45 | 0.55 | 0.65 |
| 4.92 | 0.28 | 0.39 | 0.51 | 0.46 | 0.55 | 0.65 |
| 5.08 | 0.29 | 0.40 | 0.51 | 0.47 | 0.56 | 0.65 |
| 5.24 | 0.30 | 0.41 | 0.52 | 0.47 | 0.56 | 0.65 |
| 5.40 | 0.32 | 0.42 | 0.52 | 0.48 | 0.56 | 0.65 |
| 5.56 | 0.32 | 0.42 | 0.53 | 0.48 | 0.56 | 0.64 |
| 5.72 | 0.33 | 0.43 | 0.53 | 0.49 | 0.56 | 0.64 |
| 5.88 | 0.34 | 0.44 | 0.53 | 0.49 | 0.57 | 0.64 |
| 6.04 | 0.34 | 0.44 | 0.54 | 0.49 | 0.57 | 0.64 |
| 6.20 | 0.35 | 0.44 | 0.54 | 0.49 | 0.56 | 0.64 |
| 6.36 | 0.35 | 0.45 | 0.54 | 0.49 | 0.56 | 0.64 |
| 6.52 | 0.36 | 0.45 | 0.55 | 0.48 | 0.56 | 0.64 |
| 6.68 | 0.36 | 0.45 | 0.55 | 0.48 | 0.56 | 0.63 |
| 6.84 | 0.36 | 0.45 | 0.55 | 0.48 | 0.56 | 0.63 |
| 7.00 | 0.36 | 0.46 | 0.55 | 0.48 | 0.55 | 0.63 |
| 7.16 | 0.36 | 0.46 | 0.55 | 0.47 | 0.55 | 0.62 |
| 7.32 | 0.36 | 0.46 | 0.55 | 0.47 | 0.54 | 0.62 |
| 7.48 | 0.36 | 0.45 | 0.55 | 0.47 | 0.54 | 0.61 |
| 7.64 | 0.36 | 0.45 | 0.55 | 0.46 | 0.53 | 0.61 |
| 7.80 | 0.36 | 0.45 | 0.54 | 0.46 | 0.53 | 0.60 |
| 7.96 | 0.36 | 0.45 | 0.54 | 0.45 | 0.52 | 0.59 |
| 8.12 | 0.36 | 0.45 | 0.54 | 0.45 | 0.52 | 0.59 |
| 8.28 | 0.36 | 0.45 | 0.53 | 0.44 | 0.51 | 0.58 |
| 8.44 | 0.35 | 0.44 | 0.53 | 0.43 | 0.50 | 0.57 |
| 8.60 | 0.35 | 0.44 | 0.52 | 0.43 | 0.50 | 0.56 |
| 8.76 | 0.35 | 0.43 | 0.52 | 0.42 | 0.49 | 0.55 |
| 8.92 | 0.35 | 0.43 | 0.51 | 0.42 | 0.48 | 0.55 |

| Age | Inattention-Externalizing | | | Hyperactivity/Impulsivity-Externalizing | | |
|---|---|---|---|---|---|---|
| | 95% CI_LL | Intercept | 95% CI_UL | 95% CI_LL | Intercept | 95% CI_UL |
| 9.08 | 0.34 | 0.43 | 0.51 | 0.41 | 0.47 | 0.54 |
| 9.24 | 0.34 | 0.42 | 0.50 | 0.40 | 0.47 | 0.53 |
| 9.40 | 0.34 | 0.42 | 0.50 | 0.40 | 0.46 | 0.52 |
| 9.56 | 0.33 | 0.41 | 0.49 | 0.39 | 0.45 | 0.51 |
| 9.72 | 0.33 | 0.41 | 0.49 | 0.38 | 0.44 | 0.51 |
| 9.88 | 0.32 | 0.40 | 0.48 | 0.37 | 0.43 | 0.50 |
| 10.04 | 0.32 | 0.40 | 0.48 | 0.36 | 0.43 | 0.49 |
| 10.20 | 0.31 | 0.39 | 0.47 | 0.36 | 0.42 | 0.48 |
| 10.36 | 0.31 | 0.39 | 0.47 | 0.35 | 0.41 | 0.48 |
| 10.52 | 0.30 | 0.38 | 0.46 | 0.34 | 0.40 | 0.47 |
| 10.68 | 0.29 | 0.37 | 0.46 | 0.33 | 0.40 | 0.46 |
| 10.84 | 0.28 | 0.37 | 0.45 | 0.32 | 0.39 | 0.46 |
| 11.00 | 0.28 | 0.36 | 0.45 | 0.31 | 0.38 | 0.45 |
| 11.16 | 0.27 | 0.36 | 0.45 | 0.30 | 0.37 | 0.45 |
| 11.32 | 0.26 | 0.35 | 0.44 | 0.29 | 0.37 | 0.44 |
| 11.48 | 0.25 | 0.35 | 0.44 | 0.28 | 0.36 | 0.44 |
| 11.64 | 0.25 | 0.34 | 0.44 | 0.27 | 0.35 | 0.43 |
| 11.80 | 0.24 | 0.34 | 0.43 | 0.27 | 0.35 | 0.43 |
| 11.96 | 0.23 | 0.33 | 0.43 | 0.26 | 0.34 | 0.42 |
| 12.12 | 0.22 | 0.33 | 0.43 | 0.25 | 0.34 | 0.42 |
| 12.28 | 0.22 | 0.32 | 0.42 | 0.24 | 0.33 | 0.42 |
| 12.44 | 0.21 | 0.32 | 0.42 | 0.23 | 0.33 | 0.42 |
| 12.60 | 0.20 | 0.31 | 0.42 | 0.23 | 0.32 | 0.41 |
| 12.76 | 0.20 | 0.31 | 0.42 | 0.22 | 0.32 | 0.41 |
| 12.92 | 0.19 | 0.30 | 0.41 | 0.22 | 0.31 | 0.41 |
| 13.08 | 0.19 | 0.30 | 0.41 | 0.21 | 0.31 | 0.41 |
| 13.24 | 0.18 | 0.30 | 0.41 | 0.21 | 0.31 | 0.41 |
| 13.40 | 0.18 | 0.29 | 0.41 | 0.20 | 0.30 | 0.41 |
| 13.56 | 0.18 | 0.29 | 0.41 | 0.20 | 0.30 | 0.41 |
| 13.72 | 0.17 | 0.29 | 0.41 | 0.20 | 0.30 | 0.41 |
| 13.88 | 0.17 | 0.29 | 0.41 | 0.20 | 0.30 | 0.41 |
| 14.04 | 0.17 | 0.29 | 0.41 | 0.20 | 0.30 | 0.41 |
| 14.20 | 0.16 | 0.29 | 0.41 | 0.20 | 0.30 | 0.41 |
| 14.36 | 0.16 | 0.29 | 0.41 | 0.20 | 0.30 | 0.41 |
| 14.52 | 0.16 | 0.29 | 0.41 | 0.20 | 0.31 | 0.41 |
| 14.68 | 0.16 | 0.29 | 0.41 | 0.20 | 0.31 | 0.42 |
| 14.84 | 0.16 | 0.29 | 0.42 | 0.20 | 0.31 | 0.42 |
| 15.00 | 0.16 | 0.29 | 0.42 | 0.21 | 0.32 | 0.43 |
| 15.16 | 0.16 | 0.29 | 0.43 | 0.21 | 0.32 | 0.43 |

| Age | Inattention-Externalizing | | | Hyperactivity/Impulsivity-Externalizing | | |
|---|---|---|---|---|---|---|
| | 95% CI_LL | Intercept | 95% CI_UL | 95% CI_LL | Intercept | 95% CI_UL |
| 15.32 | 0.15 | 0.29 | 0.43 | 0.22 | 0.33 | 0.44 |
| 15.48 | 0.15 | 0.30 | 0.44 | 0.22 | 0.34 | 0.45 |
| 15.64 | 0.15 | 0.30 | 0.46 | 0.23 | 0.34 | 0.46 |
| 15.80 | 0.15 | 0.31 | 0.47 | 0.24 | 0.35 | 0.47 |
| 15.96 | 0.14 | 0.31 | 0.48 | 0.24 | 0.36 | 0.48 |
| 16.12 | 0.14 | 0.32 | 0.50 | 0.25 | 0.37 | 0.49 |
| 16.28 | 0.13 | 0.33 | 0.52 | 0.26 | 0.38 | 0.51 |
| 16.44 | 0.13 | 0.33 | 0.54 | 0.27 | 0.40 | 0.52 |
| 16.60 | 0.12 | 0.34 | 0.57 | 0.28 | 0.41 | 0.54 |
| 16.76 | 0.11 | 0.35 | 0.59 | 0.29 | 0.43 | 0.56 |
| 16.92 | 0.10 | 0.36 | 0.62 | 0.29 | 0.44 | 0.59 |
| 17.08 | 0.09 | 0.37 | 0.65 | 0.30 | 0.46 | 0.61 |
| 17.24 | 0.08 | 0.39 | 0.69 | 0.31 | 0.48 | 0.64 |
| 17.40 | 0.07 | 0.40 | 0.72 | 0.32 | 0.49 | 0.67 |
| 17.56 | 0.06 | 0.41 | 0.76 | 0.33 | 0.52 | 0.70 |
| 17.72 | 0.05 | 0.43 | 0.80 | 0.34 | 0.54 | 0.74 |
| 17.88 | 0.04 | 0.44 | 0.85 | 0.35 | 0.56 | 0.77 |
| 18.04 | 0.03 | 0.46 | 0.89 | 0.35 | 0.58 | 0.81 |
| 18.20 | 0.01 | 0.48 | 0.94 | 0.36 | 0.61 | 0.86 |
| 18.36 | 0.00 | 0.50 | 0.99 | 0.37 | 0.64 | 0.90 |
| 18.52 | -0.02 | 0.52 | 1.05 | 0.38 | 0.66 | 0.95 |
| 18.68 | -0.03 | 0.54 | 1.11 | 0.39 | 0.69 | 1.00 |
| 18.84 | -0.05 | 0.56 | 1.17 | 0.40 | 0.72 | 1.05 |

*Note.* TVEM = time-varying effect modeling. ADHD = attention deficit/hyperactivity disorder. CI = confidence interval. LL = lower limit. UL = upper limit.

Table S7. Model Fit Comparison Across Random Effect Specifications

| Model | Random Effects Specification | AIC | ΔAIC (vs simpler model) | BIC | ΔBIC (vs simpler model) | Likelihood Ratio | *p* value |
|---|---|---|---|---|---|---|---|
| **Inattention-Internalizing** | | | | | | | |
| Inattention-Internalizing Baseline | Participant only | 5110.580 | - | 5146.780 | - | - | - |
| Inattention-Internalizing Baseline | Participant + Site | 5108.920 | 1.660 | 5149.640 | -2.870 | 3.660 | 0.056 |
| Inattention-Internalizing Moderated by Sex | Participant only | 5083.080 | - | 5132.790 | - | - | - |
| Inattention-Internalizing Moderated by Sex | Participant + Site | 5081.470 | 1.610 | 5135.700 | -2.910 | 3.610 | 0.057 |
| Inattention-Internalizing Moderated by Parental Education | Participant only | 4104.150 | - | 4151.560 | - | - | - |
| Inattention-Internalizing Moderated by Parental Education | Participant + Site | 4103.410 | 0.740 | 4155.130 | -3.570 | 2.740 | 0.098 |
| Inattention-Internalizing Moderated by NF1 Inheritance Mode | Participant only | 4226.550 | - | 4274.220 | - | - | - |
| Inattention-Internalizing Moderated by NF1 Inheritance Mode | Participant + Site | 4225.490 | 1.060 | 4277.490 | -3.270 | 3.060 | 0.080 |
| **Inattention-Externalizing** | | | | | | | |
| Inattention-Externalizing Baseline | Participant only | 4882.470 | - | 4918.670 | - | - | - |
| Inattention-Externalizing Baseline | Participant + Site | 4884.470 | -2.000 | 4925.190 | -6.530 | 0.000 | 1.000 |
| Inattention-Externalizing Moderated by Sex | Participant only | 4855.600 | - | 4905.310 | - | - | - |
| Inattention-Externalizing Moderated by Sex | Participant + Site | 4857.000 | -1.400 | 4911.230 | -5.920 | 0.600 | 0.437 |
| Inattention-Externalizing Moderated by Parental Education | Participant only | 3873.750 | - | 3921.160 | - | - | - |
| Inattention-Externalizing Moderated by Parental Education | Participant + Site | 3875.840 | -2.090 | 3927.560 | -6.400 | -0.090 | 1.000 |
| Inattention-Externalizing Moderated by NF1 Inheritance Mode | Participant only | 4010.390 | - | 4058.060 | - | - | -- |
| Inattention-Externalizing Moderated by NF1 Inheritance Mode | Participant + Site | 4012.440 | -2.050 | 4064.440 | -6.380 | -0.050 | 1.000 |
| **Hyperactivity/Impulsivity-Internalizing** | | | | | | | |
| Hyperactivity/Impulsivity-Internalizing Baseline | Participant only | 5135.590 | - | 5171.810 | -- | - | - |
| Hyperactivity/Impulsivity-Internalizing Baseline | Participant + Site | 5136.180 | -0.590 | 5176.930 | -5.120 | 1.410 | 0.235 |
| Hyperactivity/Impulsivity-Internalizing Moderated by Sex | Participant only | 5105.810 | - | 5155.550 | -- | -- | -- |
| Hyperactivity/Impulsivity-Internalizing Moderated by Sex | Participant + Site | 5106.590 | -0.780 | 5160.850 | -5.300 | 1.220 | 0.270 |

| | | | | | | | |
|---|---|---|---|---|---|---|---|
| Hyperactivity/Impulsivity-Internalizing Moderated by Parental Education | Participant only | 4135.540 | - | 4183.010 | - | - | - |
| Hyperactivity/Impulsivity-Internalizing Moderated by Parental Education | Participant + Site | 4135.110 | 0.440 | 4186.890 | -3.880 | 2.440 | 0.118 |
| Hyperactivity/Impulsivity-Internalizing Moderated by NF1 Inheritance Mode | Participant only | 4251.450 | -- | 4299.160 | - | - | - |
| Hyperactivity/Impulsivity-Internalizing Moderated by NF1 Inheritance Mode | Participant + Site | 4251.840 | -0.390 | 4303.890 | -4.730 | 1.610 | 0.204 |
| **Hyperactivity/Impulsivity-Externalizing** | | | | | | | |
| Hyperactivity/Impulsivity-Externalizing Baseline | Participant only | 4827.740 | - | 4863.970 | - | - | - |
| Hyperactivity/Impulsivity-Externalizing Baseline | Participant + Site | 4829.480 | -1.730 | 4870.230 | -6.260 | 0.270 | 0.603 |
| Hyperactivity/Impulsivity-Externalizing Moderated by Sex | Participant only | 4799.580 | - | 4849.320 | - | - | - |
| Hyperactivity/Impulsivity-Externalizing Moderated by Sex | Participant + Site | 4801.590 | -2.010 | 4855.850 | -6.530 | -0.010 | 1.000 |
| Hyperactivity/Impulsivity-Externalizing Moderated by Parental Education | Participant only | 3857.940 | - | 3905.410 | - | - | - |
| Hyperactivity/Impulsivity-Externalizing Moderated by Parental Education | Participant + Site | 3859.830 | -1.890 | 3911.620 | -6.200 | 0.110 | 0.737 |
| Hyperactivity/Impulsivity-Externalizing Moderated by NF1 Inheritance Mode | Participant only | 3981.490 | - | 4029.190 | - | - | - |
| Hyperactivity/Impulsivity-Externalizing Moderated by NF1 Inheritance Mode | Participant + Site | 3982.510 | -1.020 | 4034.550 | -5.360 | 0.980 | 0.322 |

Figure S1. Age-Varying Associations between ADHD Symptoms and Internalizing Problems Moderated by Sex

A. Interaction term of inattention and sex

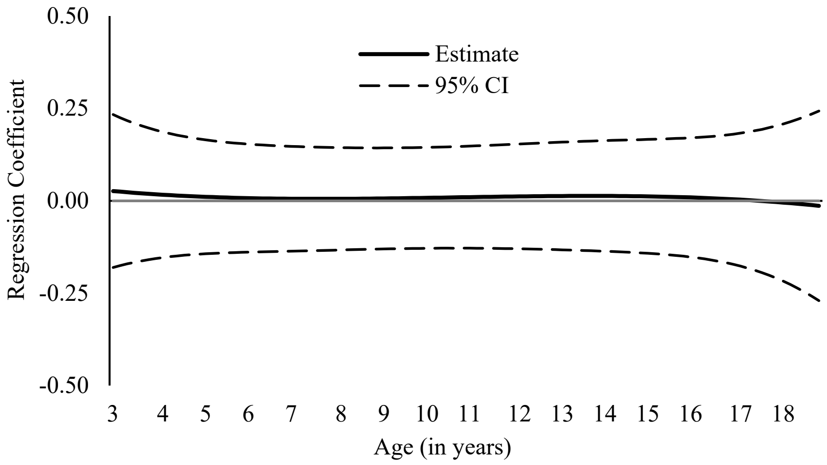


B. Interaction term of hyperactivity/impulsivity and sex

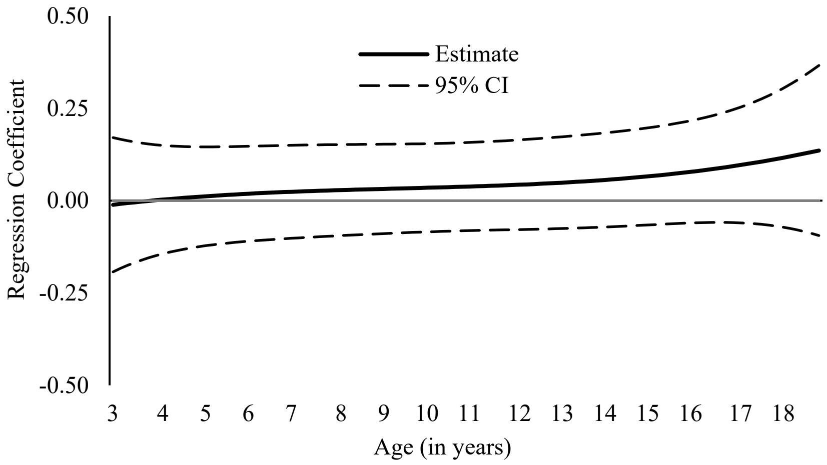


*Note*. ADHD = attention deficit/hyperactivity disorder. A significant association is indicated by 95% confidence intervals (CIs) that do not include 0. Significant age differences are indicated by non-overlapping 95% CIs between specific age points.

Figure S2. Age-Varying Associations between ADHD Symptoms and Internalizing Problems Moderated by Parental Education

A. Interaction term of inattention and parental education

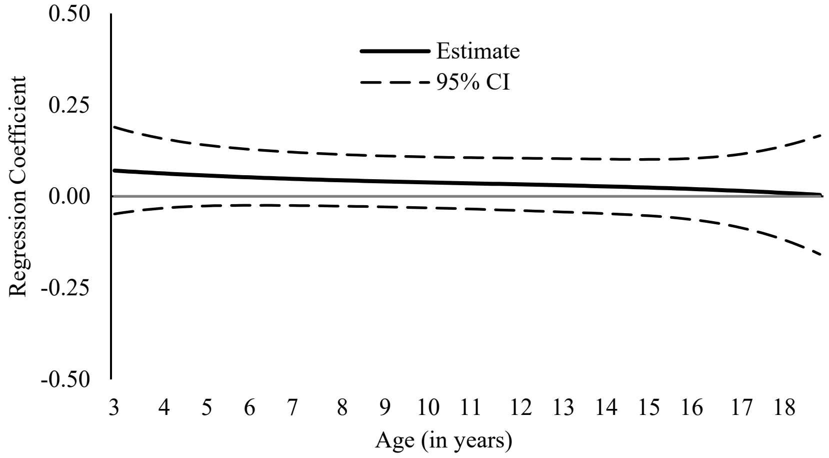


B. Interaction term of hyperactivity/impulsivity and parental education

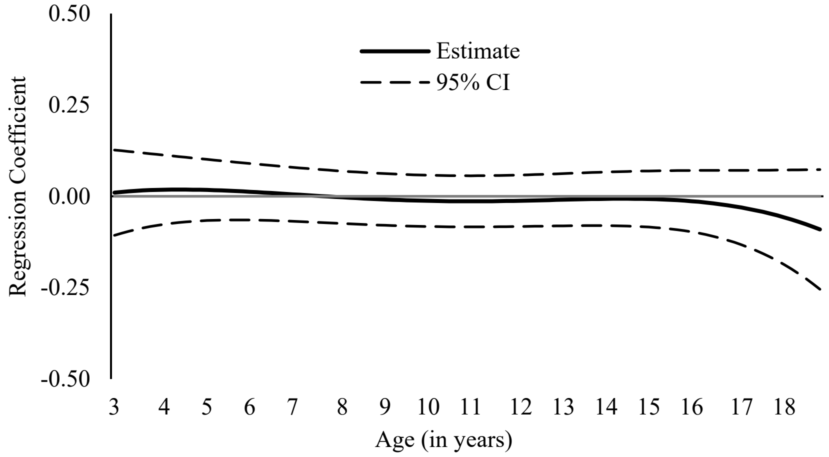


*Note*. ADHD = attention deficit/hyperactivity disorder. A significant association is indicated by 95% confidence intervals (CIs) that do not include 0. Significant age differences are indicated by non-overlapping 95% CIs between specific age points.

Figure S3. Age-Varying Associations between ADHD Symptoms and Internalizing Problems Moderated by Mode of NF1 Inheritance

A. Interaction term of inattention and mode of NF1 inheritance

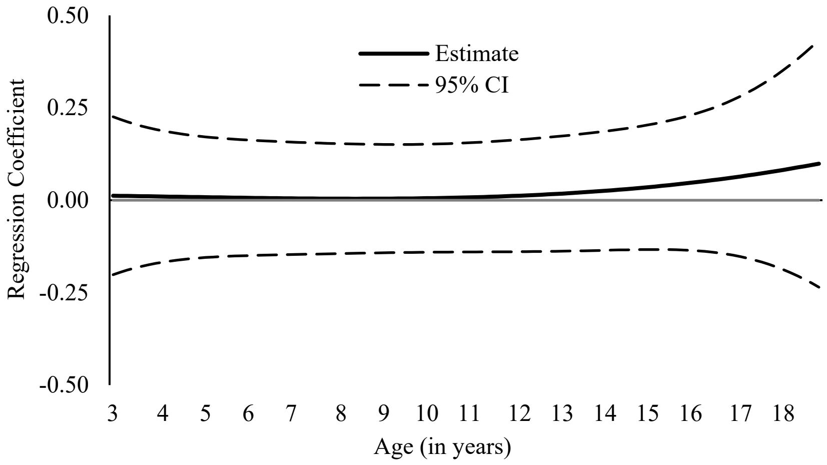


B. Interaction term of hyperactivity/impulsivity and mode of NF1 inheritance

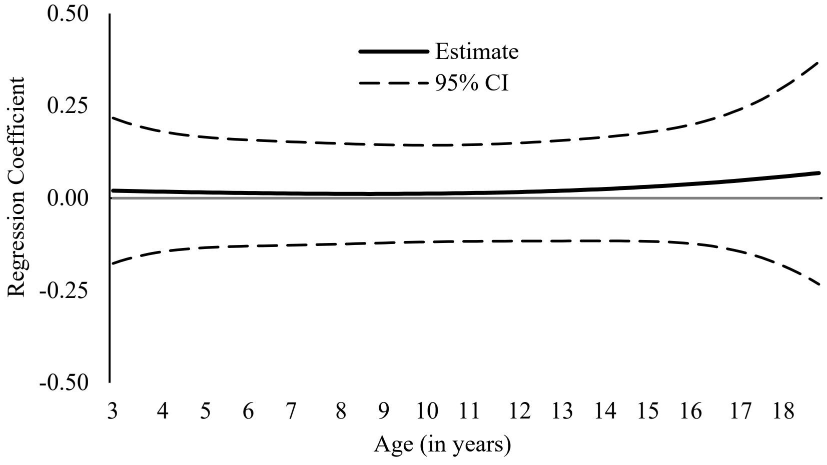


*Note*. ADHD = attention deficit/hyperactivity disorder. NF1 = neurofibromatosis type 1. A significant association is indicated by 95% confidence intervals (CIs) that do not include 0. Significant age differences are indicated by non-overlapping 95% CIs between specific age points.

Figure S4. Age-Varying Associations between ADHD Symptoms and Externalizing Problems Moderated by Sex

A. Interaction term of inattention and sex

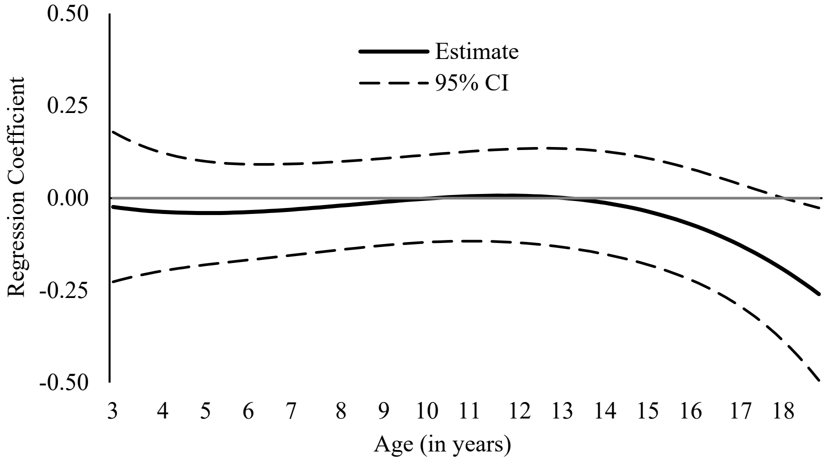


B. Interaction term of hyperactivity/impulsivity and sex

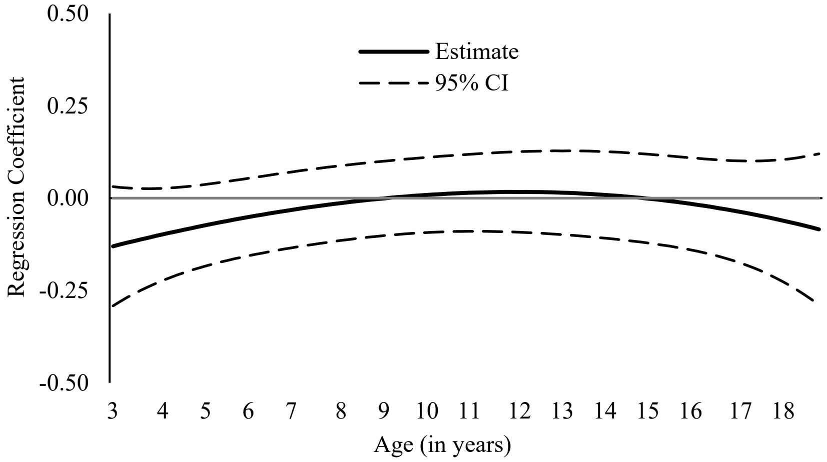


*Note*. ADHD = attention deficit/hyperactivity disorder. A significant association is indicated by 95% confidence intervals (CIs) that do not include 0. Significant age differences are indicated by non-overlapping 95% CIs between specific age points.

Figure S5. Age-Varying Associations between ADHD Symptoms and Externalizing Problems Moderated by Parental Education

A. Interaction term of inattention and parental education

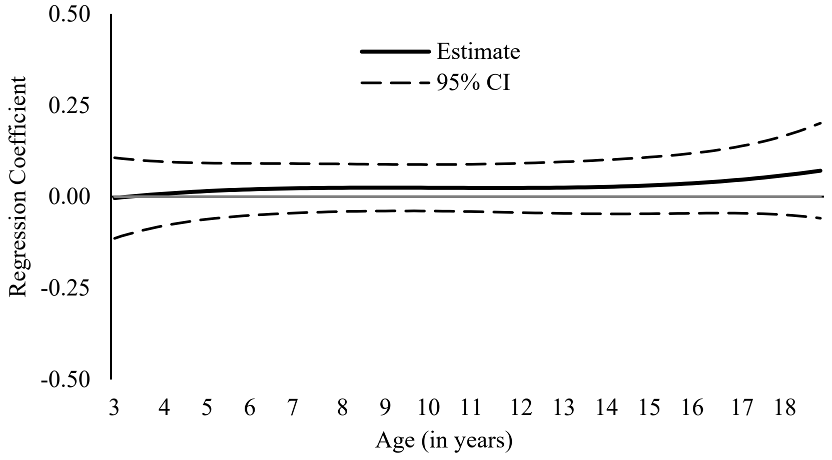


B. Interaction term of hyperactivity/impulsivity and parental education

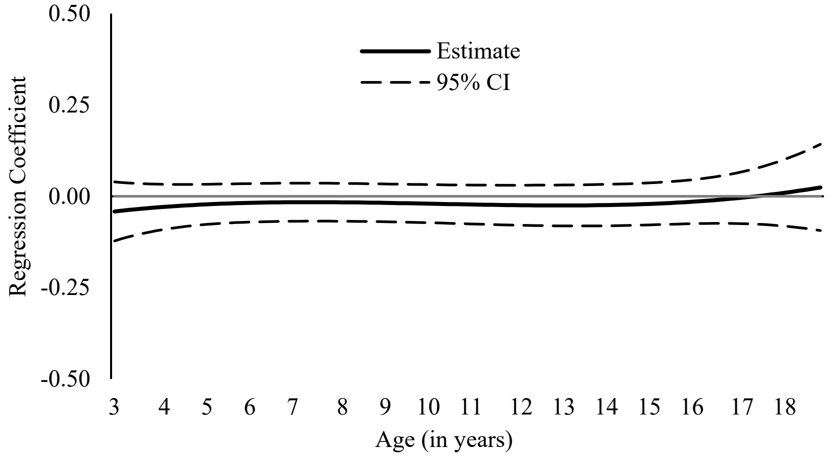


*Note*. ADHD = attention deficit/hyperactivity disorder. A significant association is indicated by 95% confidence intervals (CIs) that do not include 0. Significant age differences are indicated by non-overlapping 95% CIs between specific age points.

Figure S6. Age-Varying Associations between ADHD Symptoms and Externalizing Problems Moderated by Mode of NF1 Inheritance

A. Interaction term of inattention and mode of NF1 inheritance

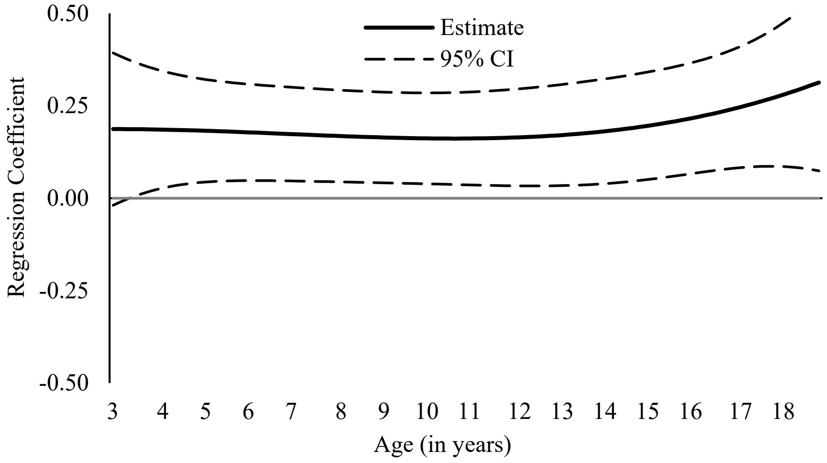


B. Interaction term of hyperactivity/impulsivity and mode of NF1 inheritance

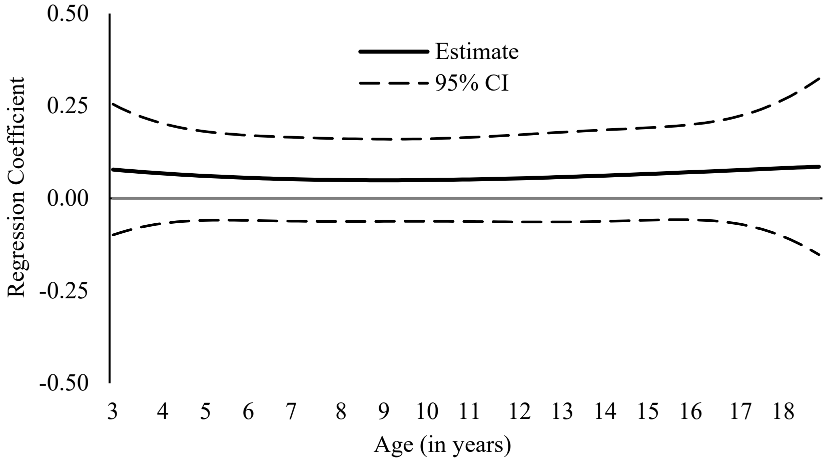


*Note*. ADHD = attention deficit/hyperactivity disorder. NF1 = neurofibromatosis type 1. A significant association is indicated by 95% confidence intervals (CIs) that do not include 0. Significant age differences are indicated by non-overlapping 95% CIs between specific age points.

Figure S7. Age-Varying Associations between ADHD Symptoms and Internalizing Problems Controlling for CBCL or BASC Instrument Type

A. Inattention-Internalizing

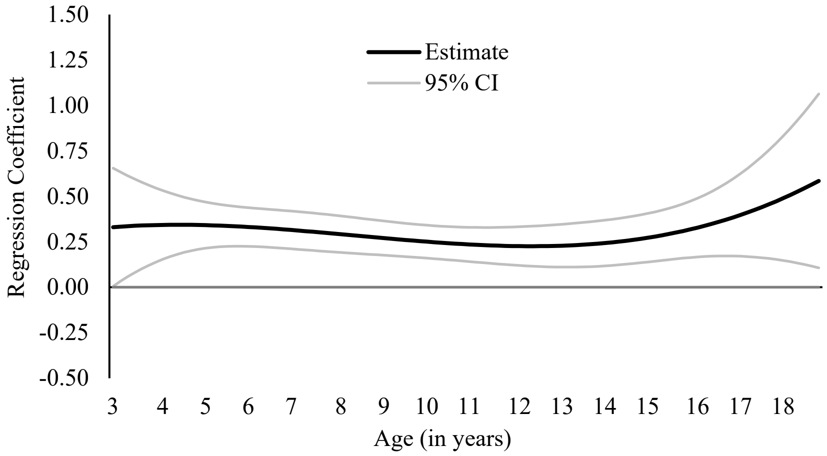


B. Hyperactivity/Impulsivity-Internalizing

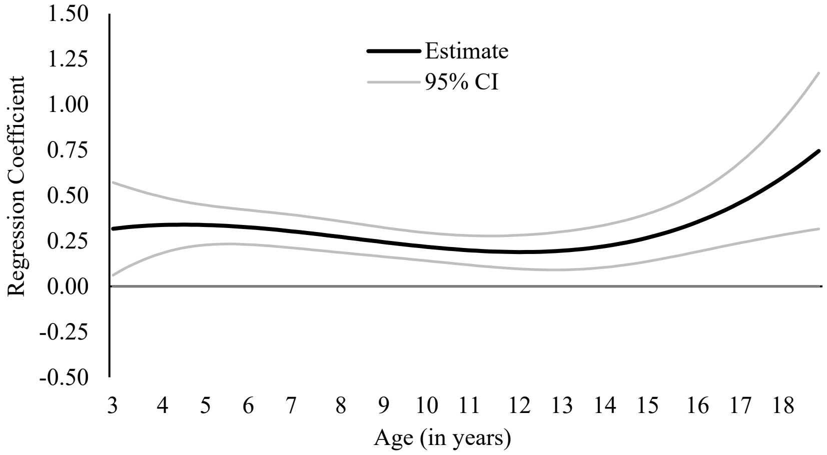


*Note*. ADHD = attention deficit/hyperactivity disorder. CBCL = Child Behavior Checklist. BASC = Behavior Assessment System for Children. The x-axis represents age, and the y-axis represents regression coefficient estimates based on T-score–scaled (*M* = 50, *SD* = 10) predictor and outcome. A significant association is indicated by 95% confidence intervals (CIs) that do not include 0. Significant age differences are indicated by non-overlapping 95% CIs between specific age points.

Figure S8. Age-Varying Associations between ADHD Symptoms and Externalizing Problems Controlling for CBCL or BASC Instrument Type

A. Inattention-Externalizing

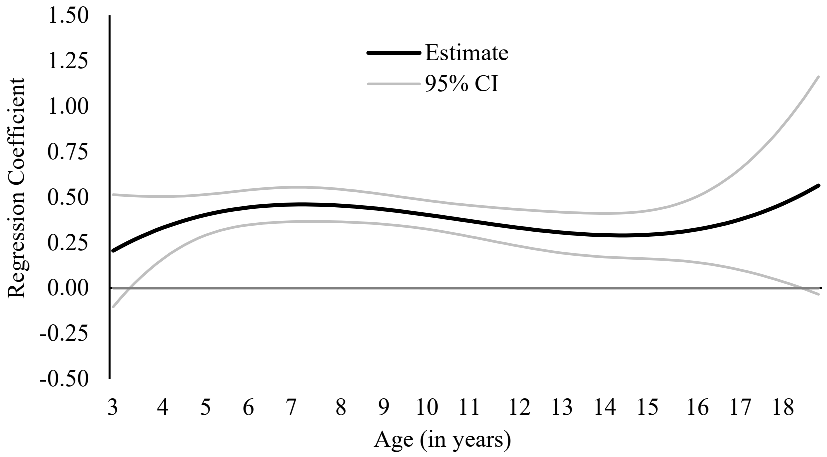


B. Hyperactivity/Impulsivity-Externalizing

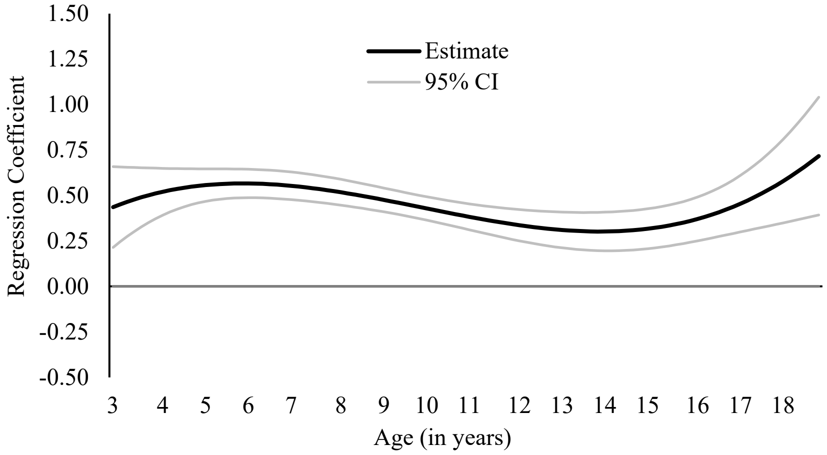


*Note*. ADHD = attention deficit/hyperactivity disorder. CBCL = Child Behavior Checklist. BASC = Behavior Assessment System for Children. The x-axis represents age, and the y-axis represents regression coefficient estimates based on T-score–scaled (*M* = 50, *SD* = 10) predictor and outcome. A significant association is indicated by 95% confidence intervals (CIs) that do not include 0. Significant age differences are indicated by non-overlapping 95% CIs between specific age points.
